# Design, synthesis and cytotoxic research of a novel antitumor model based on acrylamide–PABA analogs *via* β-tubulin inhibition†

**DOI:** 10.1039/d5ra02384j

**Published:** 2025-06-03

**Authors:** Maha Ali Alghamdi, Mustafa R. Abdulbaqi, Dalal Sulaiman Alshaya, Jawaher Alharthi, Hanadi A. Katouah, Fahmy Gad Elsaid, Eman Fayad, Ali H. Abu Almaaty, Abdullah Yahya Abdullah Alzahrani, Botros Y. Beshay

**Affiliations:** a Department of Biotechnology, College of Sciences, Taif University P.O. Box 11099 Taif 21944 Saudi Arabia; b Department of Pharmaceutics, College of Pharmacy, Al-Naji University Baghdad 10015 Iraq; c Department of Biology, College of Science, Princess Nourah bint Abdulrahman University P.O. Box 84428 Riyadh 11671 Saudi Arabia; d Chemistry Department, College of Science, Umm Al-Qura University 21955 Makkah Saudi Arabia; e Department of Biology, College of Science, King Khalid University PO Box 960 Asir Abha 61421 Saudi Arabia; f Department of Zoology, Faculty of Science, Port Said University Port Said Egypt aliabuelmaaty8@gmail.com; g Department of Chemistry, Faculty of Science, King Khalid University Abha 61413 Saudi Arabia; h Pharmaceutical Sciences (Pharmaceutical Chemistry) Department, College of Pharmacy, Arab Academy for Science, Technology and Maritime Transport Alexandria P.O. Box 1029 Egypt

## Abstract

Currently, the human health is facing numerous challenges, especially with regard to cancer. Therefore, new treatment options that specifically target tumor cells will inevitably improve the therapeutic toolkit for various cancers. In this regard, a sequence of acrylamide–PABA hybrids 4a–j was synthesized, and their formation was confirmed *via* spectral and elemental analyses. The compounds were evaluated for their antiproliferative activity against MCF-7 (breast), HepG2 (liver) and a normal health breast cell line (MCF-10A). Among the series, acrylamide–PABA analog 4j with a furan group in the acrylamide moiety was the most potent anti-proliferative member with a notable IC_50_ value (IC_50_ = 1.83 μM) against MCF-7 cells. Compound 4j's anti-tubulin activity and apoptosis-promoting properties were the cause of its anti-proliferative inhibitory mechanism. Compound 4j promoted apoptosis in MCF-7 cells by raising the expression of apoptotic markers, such as p53, Bax, Bcl-2 and caspase 9, with respect to the untreated control. The molecular docking study of compound 4j revealed a nice fitting into the active site of tubulin.

## Abstract 2

A new set of novel acrylamide–PABA hybrids has been designed, synthesized and screened for their *in vitro* cytotoxicity against MCF-7 (breast cancer cell line), HepG2 (liver cancer cell line) and MCF-10A (normal health breast cell line).

## Graphical abstract

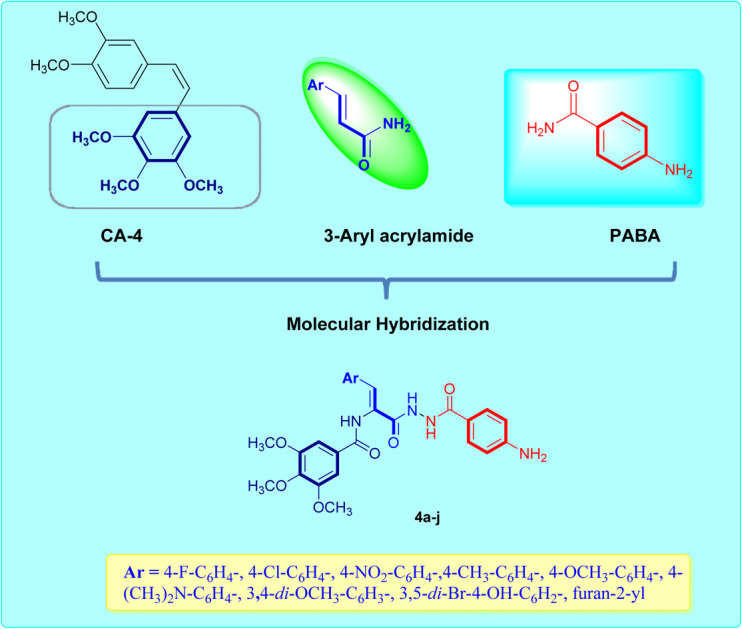

## 1. Introduction

Breast cancer represents the most commonly diagnosed lethal malignancy, and it is the leading cause of cancer-related deaths in women worldwide, presenting a critical global health issue.^1,2^ It is experienced by millions each year and is the most common cancer type occurring among women, and the second most common cancer type overall.^3^ In 2022, an estimated 2.3 million new cases and 670 000 deaths related to this disease was reported. The incidence rate is still increasing (1–5% per year).^4,5^ Hence, ongoing research focuses on unravelling the molecular mechanisms driving breast cancer to advance approaches toward its prevention, early detection, and therapeutic strategies.^6,7^

Tubulin is a dominant structural protein that is essential for the formation of microtubules, which are essential targets in breast cancer. Microtubule-targeting agents (MTAs), like taxanes (*e.g.*, paclitaxel and docetaxel) are commonly used to treat various breast cancers, including triple-negative breast cancer (TNBC).^8–10^ These drugs disrupt microtubule dynamics, stop cell division and trigger apoptosis. However, resistance to the existing MTAs has spurred the development of new tubulin inhibitors that target complementary binding sites, such as the colchicine-binding site, to inhibit microtubule polymerisation.^11–13^

Building on these findings, we aimed at designing potential tubulin inhibitors using a hybrid structure-based approach, focusing on acrylamide–PABA–combretastatin analogues as promising candidates. The anticancer activity of the acrylate moiety proceeds through a combined mechanism that includes β-tubulin and protein kinase inhibition.^14–16^ Moreover, with a strong antimitotic effect, CA-4 (Combretastatin A-4) binds to the colchicine site. A better binding affinity is also shown by the dimethoxybenzene moiety and other parts. CA-4 has advanced to phase II and III clinical studies.^17,18^ However, its clinical utility is limited by its pharmacokinetic parameters like low aqueous solubility, short plasma half-life, and isomerization from active *cis* to inactive trans under physiological settings.^19,20^

**Fig. 1:**
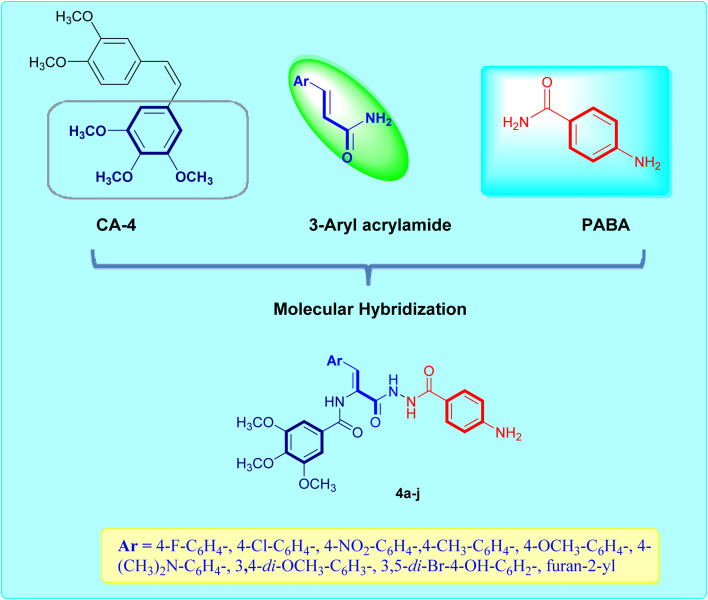
Molecular design of the target acrylamide–PABA–CA-4 hybrids.

The current study attempts to overcome the poor pharmacokinetics of CA-4 and growing resistance to the available cancer therapies through a new building block, namely, *p*-aminobenzoic acid (PABA). PABA is recognised for its structural diversity at the amino and carboxyl groups and can act as an outstanding building block. PABA-based hybrid compounds exhibit antioxidant, anti-inflammatory, anticancer and antimicrobial potential in clinical applications.^21,22^ PABA derivatives also have been demonstrated to have various modes of action in cancer chemotherapy, including the inhibition of β-tubulin and protein kinases, further supporting their potential as therapeutic agents.^23,24^ In this work, we integrated *p*-aminobenzoic acid (PABA), the acrylate platform and trimethoxybenzene moiety from CA-4 (Fig. 1) into a single molecular entity to enhance its pharmacokinetic profile, along with improved inhibition of microtubule polymerization. We have also introduced several substitution patterns on the aryl group linked to the acrylamide part to assess their impact on tubulin binding and inhibitory activity akin to colchicine. This design strategy will assess the antiproliferative potential of the newly synthesized compounds against the MCF-7 breast cancer cell line.

## 2. Results and discussion

### 2.1. Chemistry

**Scheme 1:**
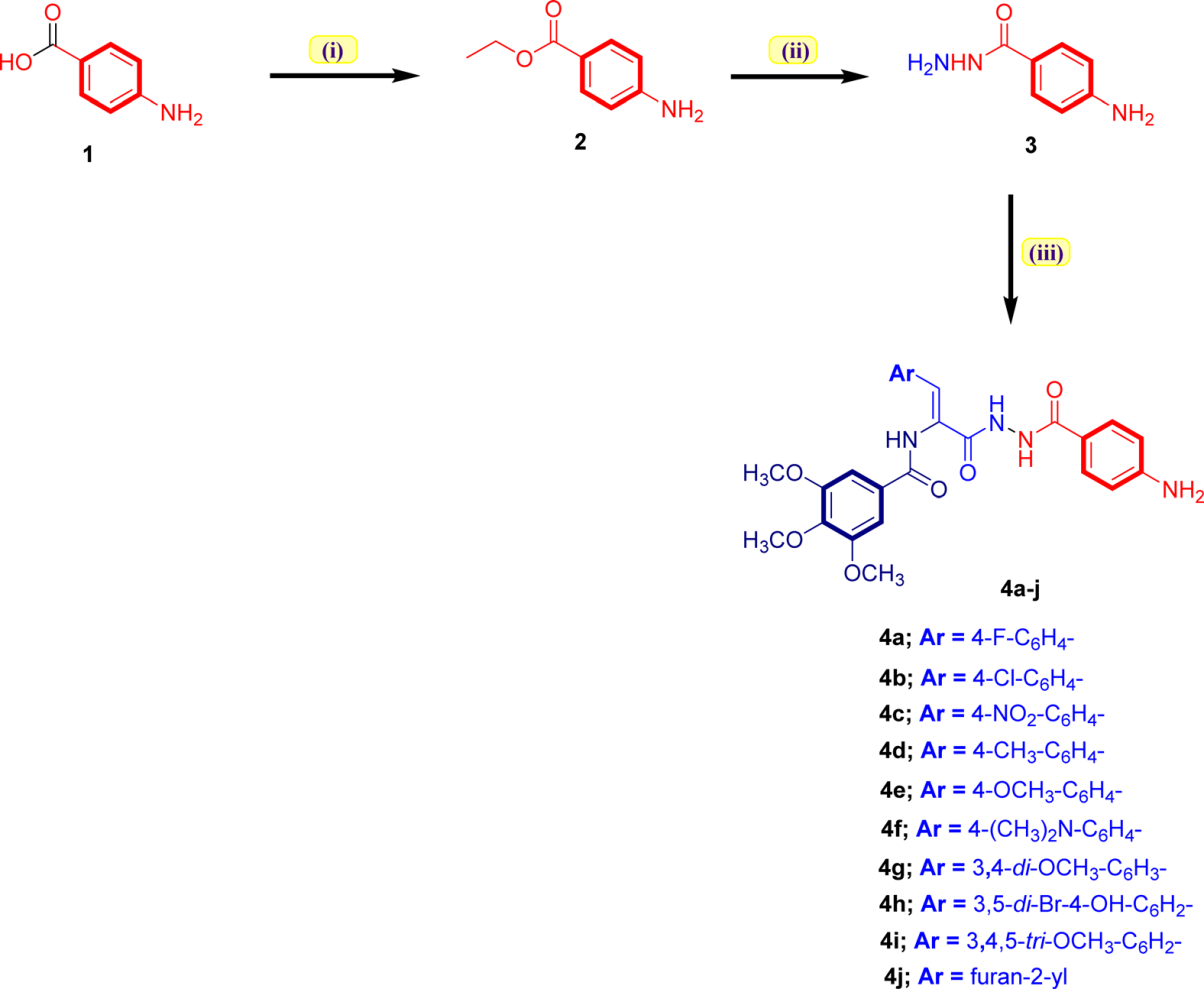
Synthesis of the targeted 3-arylacrylamide–PABA analogs 4a–j. Reaction conditions: (i) EtOH, conc. H_2_SO_4_, reflux 12 h; (ii) NH_2_NH_2_·H_2_O, EtOH, reflux 4 h; (iii) appropriate ethyl 3-arylacrylate, EtOH, AcOH, reflux 10–12 h.

The synthetic employed pathway to prepare the new acrylamide–PABA analogs 4a–j is outlined in Scheme 1. The chemical synthesis was initiated by heating 4-aminobenzoic acid (1) with pure ethanol in the presence of concentrated sulphuric acid at reflux temperature to furnish ethyl 4-aminobenzoate (2). The obtained ester 2, on heating to reflux with hydrazine hydrate in pure ethanol, gave 4-aminobenzoic acid hydrazide (3) in good yield. The target acrylamide–PABA analogs 4a–j were prepared by reaction of the key hydrazide intermediate 3 with the appropriate ethyl 3-arylacrylate molecule in refluxing pure ethanol using a catalytic amount of glacial acetic acid to yield the desired acrylamide–PABA products 4a–j. The formation of the desired acrylamide–PABA products 4a–j was evidenced by spectral data (^1^H-NMR and ^13^C-NMR spectra), in addition to elemental analysis.

As a representative example of these series, the ^1^H-NMR spectrum of 4j showed the presence of three singlet signals at *δ*_Η_ 10.01, 9.96 and 9.83 ppm, evidencing the presence of three NH amidic protons, and also the presence of two aromatic sets of C2,6-Hs and C3,5-Hs of the phenyl ring of the PABA moiety as doublet signals at *δ*_Η_ 7.65 and 6.57 ppm, respectively, with *J* value = 8.2 Hz. In addition, there are two singlet signals with two-proton integration at *δ*_Η_ 7.40 and 5.71 ppm attributed to the phenyl ring of the 3,4,5-trimethoxyphenol moiety and amino (NH_2_) protons of the *p*-aminobenzoic acid moiety. The olefinic proton was also observed as a singlet signal at *δ*_Η_ 7.24 ppm. Furthermore, the ^1^H-NMR spectrum of the acrylamide–PABA analog 4j displayed two singlet signals at 3.87 and 3.75 ppm for six- and three-protons integration, which correspond to the aliphatic methoxyls' of the 3,4,5-trimethoxyphenyl motif. ^13^C-NMR spectrum of the acrylamide–PABA analog 4j displayed three signals in the range of *δ*_C_ 166.06–164.68 ppm assigned to the amide carbonyl groups, and two characteristic aliphatic signals with two- and one-carbon integration at *δ*_C_ 60.58 and 56.53 ppm, respectively, which were ascribed to the aliphatic methoxyls of the 3,4,5-trimethoxyphenyl group. In addition, the ^13^C-NMR spectrum of the acrylamide–PABA analog 4j demonstrated all other aromatic and olefinic carbons, which appeared at their expected chemical shifts. Finally, data from the elemental analysis further confirmed the assigned structure.

### 2.2. Biology

#### 2.2.1. Cytotoxic activity of the acrylamide–PABA analogs against the MCF-7 cell line

The MTT colorimetric technique was used to evaluate the cytotoxic activity of the newly produced acrylamide–PABA hybrids 4a–j against the MCF-7 breast cancer cell line. Colchicine (Col) was utilized as a reference cytotoxic agent in this assay technique. Table 1 shows the results as an IC_50_ for each test compound. In general, most of the examined analogs had good cytotoxic activity against MCF-7 cells with IC_50_ values ranging from 1.83–73.11 μM. Among these analogs, compounds 4c, 4d and 4e showed weak activity toward the MCF-7 cell line (IC_50_ > 30 μM) compared to Col (IC_50_ = 3.54 μM). The remaining analogs showed low micromolar IC_50_ values (IC_50_ < 30 μM). In particular, compounds 4a and 4j displayed the most activity of the new compounds with IC_50_ values of 2.99 and 1.83 μM, respectively. The structure activity relationship (SAR) analysis of the aryl ring-attached acrylamide moiety indicated an influence on the antiproliferative activity. Introduction of an electron-withdrawing group such as the 4-fluorphenyl group in compound 4a or the 4-chlorophenyl group in compound 4b resulted in an increase in the antiproliferative activity with an IC_50_ value of 2.99 and 25.27 μM, respectively. However, increasing the number of electron-withdrawing substituents (such as compound 4h with dibromo substituents) increased the antiproliferative activity (IC_50_ = 19.92 μM). Moreover, upon the introduction of electron-donating groups (such as the 4-methylphenyl group in compound 4d or 4-methoxyphenyl in compound 4e), the cytotoxic efficacy was notably decreased with an IC_50_ value of 73.11 and 64.61 μM, respectively. Furthermore, the introduction of a polysubstituted phenyl ring (such as 3,4-dimethoxyphenyl in compound 4g and 3,4,5-trimethoxyphenyl in compound 4i) led to a notable increase of the cytotoxic activity with an IC_50_ value of 17.10 and 4.51 μM, respectively. Again, the cytotoxic activity was significantly increased in acrylamide–PABA 4j when the substituted phenyl ring was replaced with a heterocyclic moiety such as furan with an IC_50_ value of 1.83 μM, which was superior to Col (1.9-fold more potent than the reference drug). These results demonstrated the importance of the heterocyclic substitution in the acrylamide moiety and the activity increased in the following order: furan > polysubstituted phenyl ring > phenyl ring decorated with electron-withdrawing groups > phenyl ring having electron-donating substituents. In addition, the acrylamide–PABA compounds that demonstrated notable cytotoxic activity against MCF-7 cells were assessed further to determine their IC_50_ values against the HepG2 hepatocellular carcinoma cell line (Table 1). All examined compounds demonstrated better cytotoxic activity against HepG2 cells relative to the reference drug Col. Additionally, the MCF-10A (normal breast) cell line was used to assess the antiproliferative efficacy of the active acrylamide–PABA compounds 4a and 4j (Table 1). It is clear from the IC_50_ values that the examined active molecules showed less cytotoxicity against the normal breast cell line (MCF-10A) relative to the anticancer potential against the carcinoma cell lines, which supports the anticancer properties of the acrylamide–PABA compounds.

**Table 1:** *In vitro* cytotoxic evaluation of the constructed acrylamide–PABA analogs 4a–j against MCF-7 breast carcinoma cells^a^

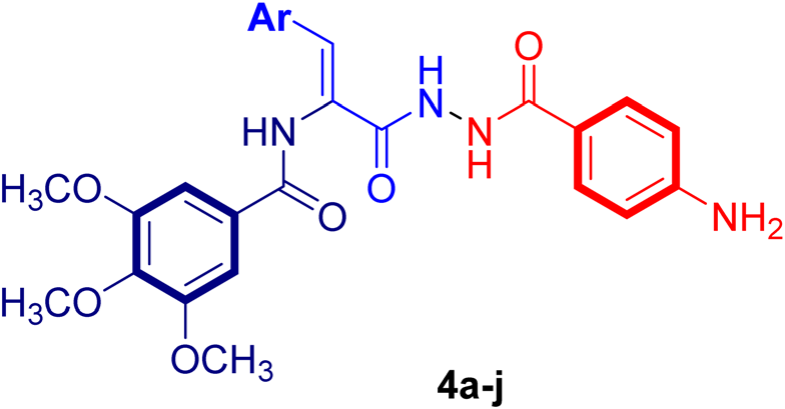				
Comp. no.	Ar	IC_50_ (μM)		
		MCF-7	HepG2	MCF-10A
4a	4-F–C_6_H_4_–	2.99 ± 0.10	4.87 ± 0.13	19.76 ± 0.62
4b	4-Cl–C_6_H_4_–	25.27 ± 0.86	ND	ND
4c	4-NO_2_–C_6_H_4_–	39.69 ± 1.34	ND	ND
4d	4-CH_3_–C_6_H_4_–	73.11 ± 2.47	ND	ND
4e	4-OCH_3_–C_6_H_4_–	64.61 ± 2.19	ND	ND
4f	4–(CH_3_)_2_N–C_6_H_4_–	27.22 ± 0.92	ND	ND
4g	3,4-di-OCH_3_–C_6_H_3_–	17.10 ± 0.58	ND	ND
4h	3,5-di-Br-4-OH–C_6_H_2_–	19.92 ± 0.67	ND	ND
4i	3,4,5-tri-OCH_3_–C_6_H_2_–	4.51 ± 0.15	ND	ND
4j	Furan-2-yl	1.83 ± 0.06	2.14 ± 0.09	23.21 ± 1.06
Col	—	3.54 ± 0.08	4.05 ± 0.07	27.54 ± 0.11

a All experiments were carried out in triplicate; ND, not determined.

#### 2.2.2. Acrylamide–PABA analogs 4a and 4j inhibited β-tubulin polymerization in MCF-7 cells

**Fig. 2:**
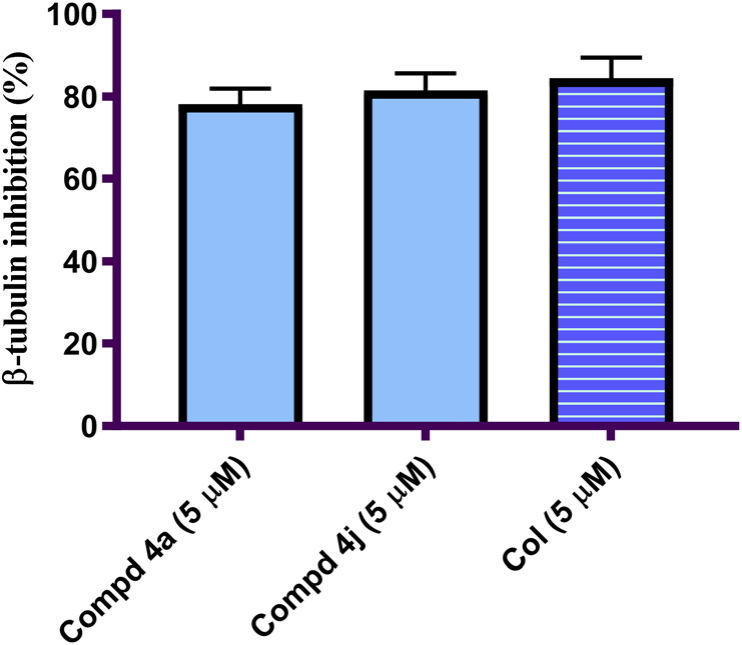
Representation of the percentage of β-tubulin polymerization on MCF-7 cells stimulated by acrylamide–PABA analogs 4a and 4j relative to Col.

The process of the tubulin-microtubule polymerization of a cancer mass usually leads to aggressive tumor growth and metastasis.^25^ This link between the abnormal tubulin assembly and tumor growth is strong owing to the excessive tubulin polymerization in more lethal cancer such as breast cancer.^26^ Dual inhibitors of cancer cell proliferation and tubulin polymerization have recently shown their remarkable potential against several cancers.^27^ The two most potent analogs 4a and 4j were next examined for their potency as β-tubulin inhibitors using the β-tubulin assay kit. Fig. 2 shows the data as the percentage inhibition for each analog using Col as a reference drug. The results indicated that the examined analogs 4a and 4j inhibited β-tubulin polymerization with a percentage inhibition of 78.19% and 81.51%, respectively, when compared to the reference Col (percentage inhibition of 84.33%). Compound 4j (Ar = furan-2yl), the most potent antiproliferative agents, displayed the greatest inhibitory efficacy against β-tubulin polymerization with a percentage inhibition of 81.51%, which was comparable to Col. On the other hand, compound 4a (Ar = 4-fluorophenyl) ranks second in β-tubulin polymerization inhibition activity with a percentage inhibition value of 78.19%. The results of this assay method indicated that acrylamide–PABA analogs 4a and 4j had substantial antiproliferative activity against MCF-7 and as potent β-tubulin inhibitors.

#### 2.2.3. Acrylamide–PABA analog 4j induced cellular apoptosis in MCF-7 cells

**Fig. 3:**
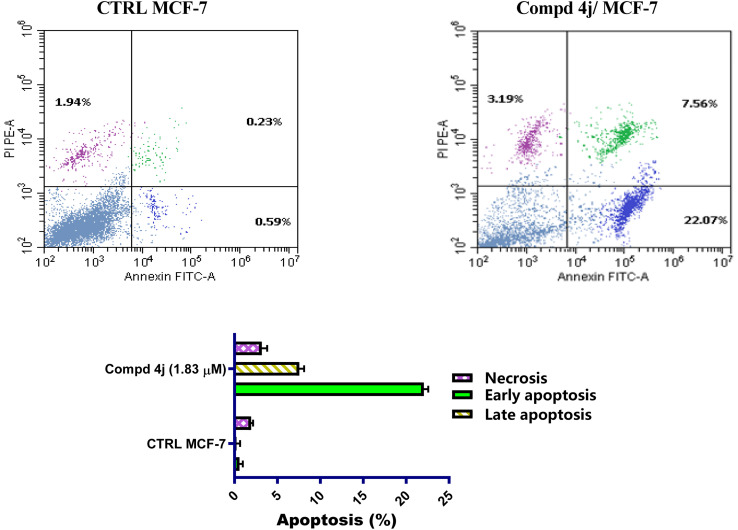
Comparison between the apoptosis influence on MCF-7 cells stimulated by acrylamide–PABA analog 4j and untreated control cells.

Understanding the biological mechanism of action of chemotherapeutic agents is an important aspect of the pharmaceutical development process.^28^ Recently, the activation of apoptosis through pharmacological manipulation of malignant cells' growth suppression and anti-proliferative action has been demonstrated as a unique approach for chemotherapeutic detection and screening.^29^ To better understand the pathway that allowed the produced analogs to elicit the cytotoxic action, we incubated the most active acrylamide–PABA 4j with MCF-7 cells for 48 h. Following cellular staining with Annexin V –FITC/propidium iodide (PI), FACS analysis was conducted. To differentiate between early and late apoptosis, both stains were used. (PI^−^/Annexin V-FITC^+^) represented early apoptotic cells, (PI^+^/Annexin V-FITC^+^) indicated late apoptotic cells, and (PI^+^/Annexin V-FITC^−^) represented necrotic cells. However, healthy cells were not stained (PI^−^/Annexin V-FITC^−^). Fig. 3 shows the dot plot of the most active analog 4j. As seen in Fig. 3, compound 4j primarily used apoptosis to exhibit its cytotoxic activity. This was clarified by compound 4j's modest degree of induced necrosis in contrast to its markedly elevated levels of induced early and late apoptosis. Acrylamide–PABA analog 4j induced both early apoptosis (22.07%) and late apoptosis (7.56%) in comparison with modest level of necrosis (3.19%). These data indicated that acrylamide–PABA analog 4j demonstrated its cytotoxic effects mainly through apoptosis.

#### 2.2.4. Acrylamide–PABA analog 4j enhanced the level of apoptotic markers in MCF-7 cells

**Fig. 4:**
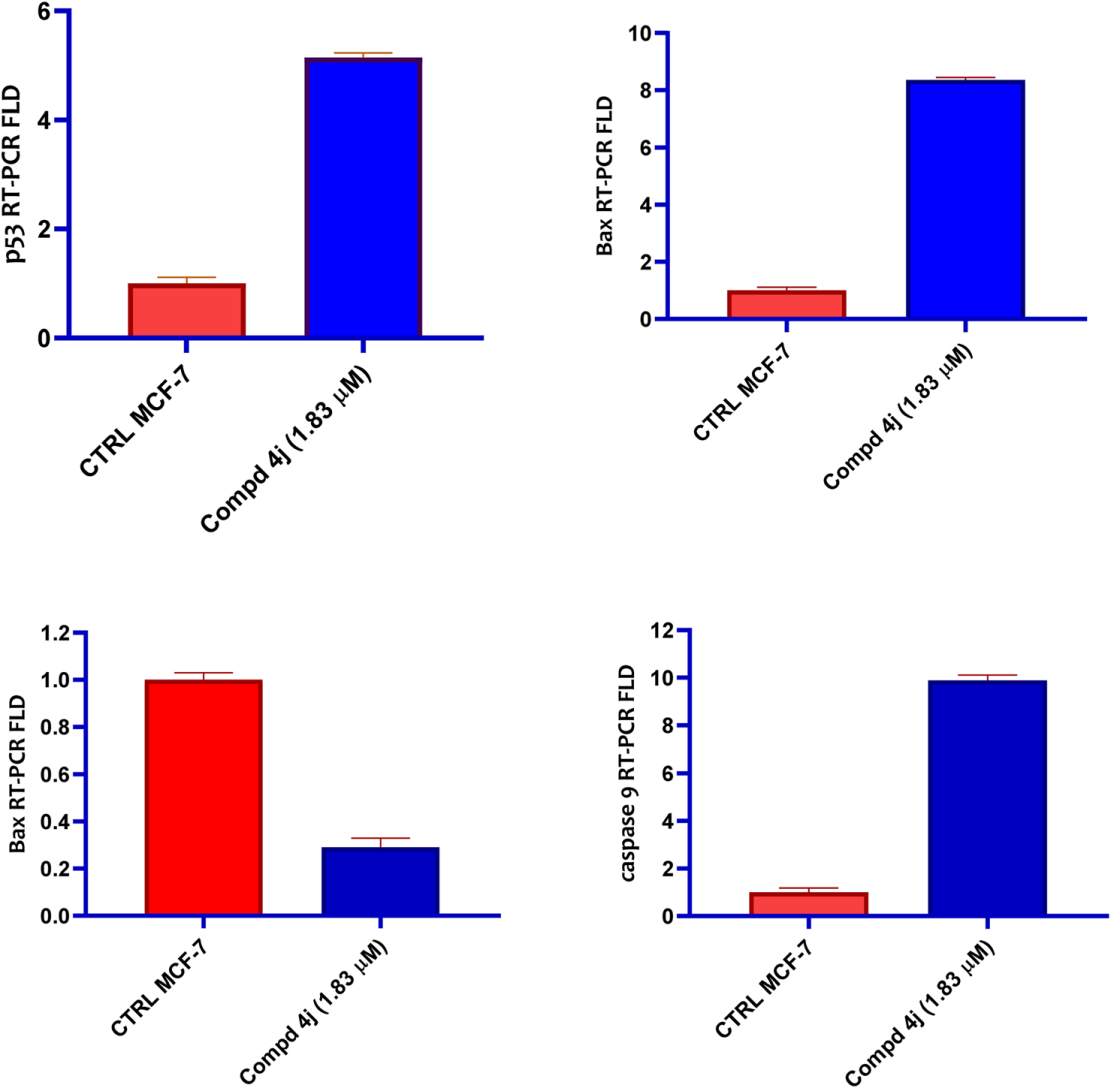
*In vitro* qRT-PCR measurements of the expression levels of p53, Bax, Bcl-2 and *c*aspase 9 in MCF-7 cells stimulated by the acrylamide–PABA analog 4j relative to negative control cells.

The apoptotic markers related to apoptosis were crucial in preventing cancer.^30^ p53 and other apoptotic regulators function as transcription factors that can activate several target genes in response to oncogenic stress, causing cell cycle arrest, apoptosis activation, and degeneration, while also promoting DNA repairs.^31^ As a result, the apoptosis-related markers work together in order to ensure that cells with mutations that promote cancer (including broken genomes) are either repaired or permanently eliminated.^32^ To further comprehend the molecular process involved in the apoptotic efficacy of the acrylamide–PABA analogs, the expression levels of p53, Bax, Bcl-2 and *c*aspase 9 were evaluated. These genes' expression levels were investigated in the presence of acrylamide–PABA analog 4j, which demonstrated significant apoptotic activity. This was carried out in contrast to a negative control group. As illustrated in Fig. 4, the acrylamide–PABA 4j analog displayed a significant elevation in the expression level of p53 (5.14-fold), Bax (8.36-fold) and *c*aspase 9 (9.89-fold) compared to the negative controls. This was accompanied by a notable drop in the level of Bcl-2 (0.29-fold). These data explained that the enhanced expression of the apoptotic markers is the main mechanism underlying the acrylamide–PABA analog 4j-induced cytotoxicity against the MCF-7 cancer cell line.

#### 2.2.5. Acrylamide–PABA analog 4j promoted cellular cycle arrest at the G2\M phase in MCF-7 cells

**Fig. 5:**
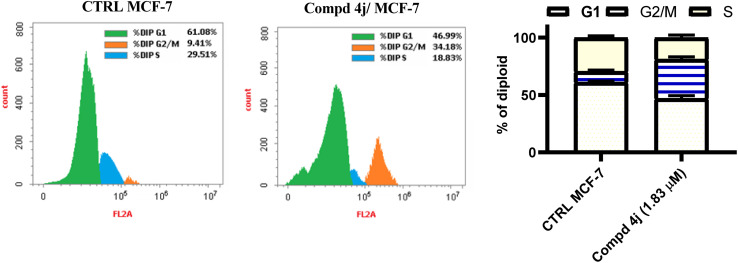
Cellular cycle distribution of MCF-7 cells stimulated by acrylamide–PABA analogs 4j relative to untreated controls.

In cancer mass development, the disruption of the cell cycle can be an important event. Cellular cycle progression has two primary checkpoints: G1/S before DNA replication and G2/M before mitosis.^33^ A p53-responsive gene such as p21^wafi^ regulates the G1/S checkpoint and mediates cell cycle arrest.^34^ Since the cytotoxic activity of the synthesized analogs against MCF-7 cells was induced by the enhanced apoptotic levels, we examined whether these analogs further induced cell cycle arrest. MCF-7 cells were treated with the most active acrylamide–PABA 4j member for 48 h, dyed with PI and then subjected to flow cytometric analysis in order examine the distribution of the cellular cycle. As can been seen in Fig. 5, the cellular distribution at each stage is depicted in the histogram of the cellular cycle caused by the acrylamide–PABA hybrid 4j. It showed that compound 4j significantly promoted cellular cycle arrest mainly at the G2/M phase (34.18%) compared with the untreated control group (9.41%). On the other hand, the analog 4j significantly decreased the cellular population at both G1 phase (46.99%) and S phase (18.83%) with regard to the negative controls [(61.08%) and (29.51%)], respectively. Hence, the promotion of the cell cycle arrest at the G2/M phase is another mechanism that explains the cytotoxicity of the acrylamide–PABA hybrid 4j against MCF-7 cells.

### 2.3. Molecular modelling study

**Fig. 6:**
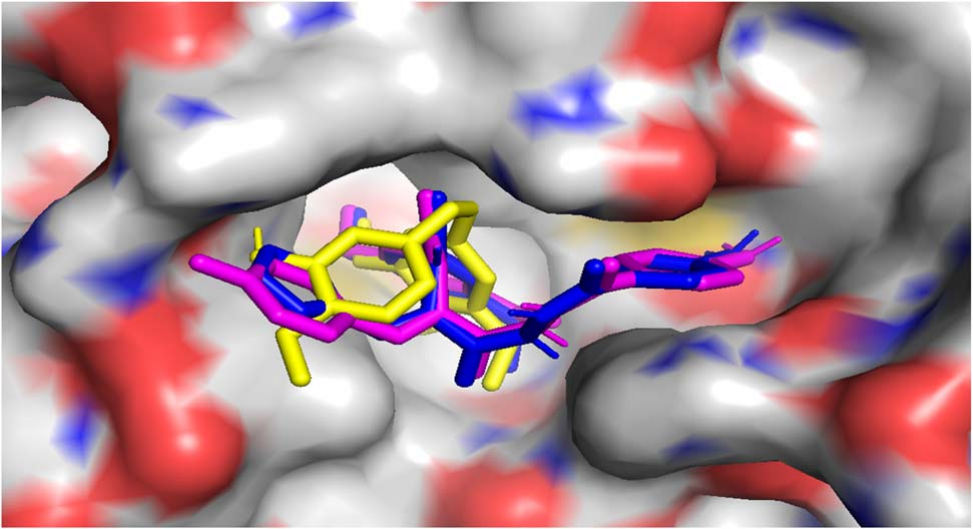
Surface view representation of the superimposition of CA-4 (yellow), 4a (magenta) and 4j (blue) in the colchicine-binding pocket of tubulin.

**Fig. 7:**
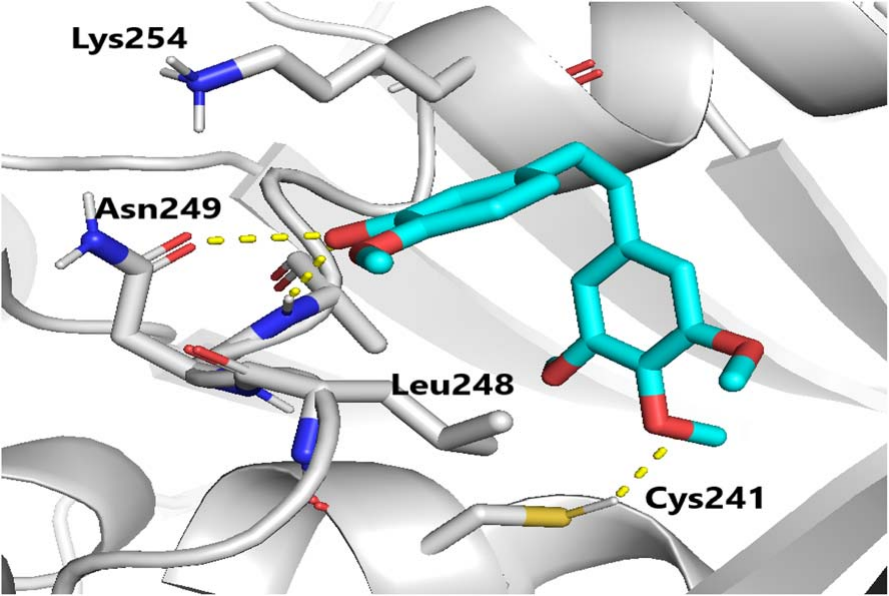
Binding mode of CA-4 in the colchicine-binding pocket of tubulin. Hydrogen bonds are shown using yellow dashed lines.

**Fig. 8:**
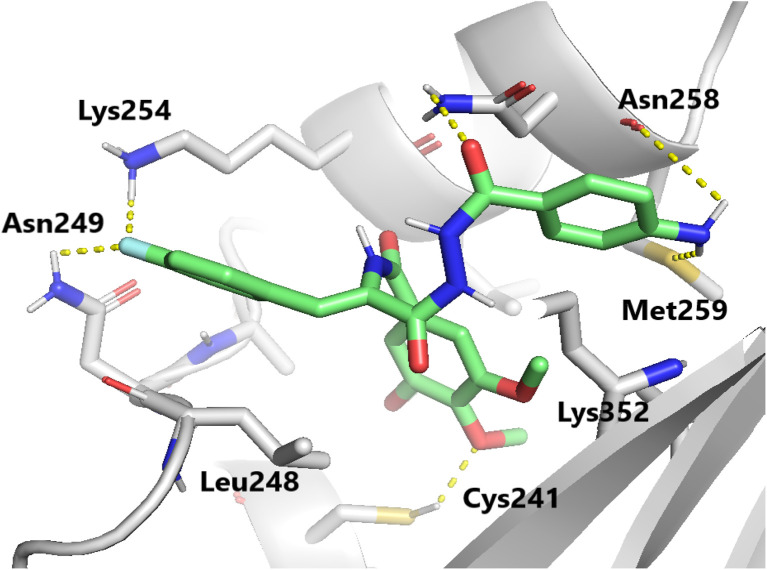
Binding mode of compound 4a in the colchicine-binding pocket of tubulin. Hydrogen bonds are shown using yellow dashed lines.

**Fig. 9:**
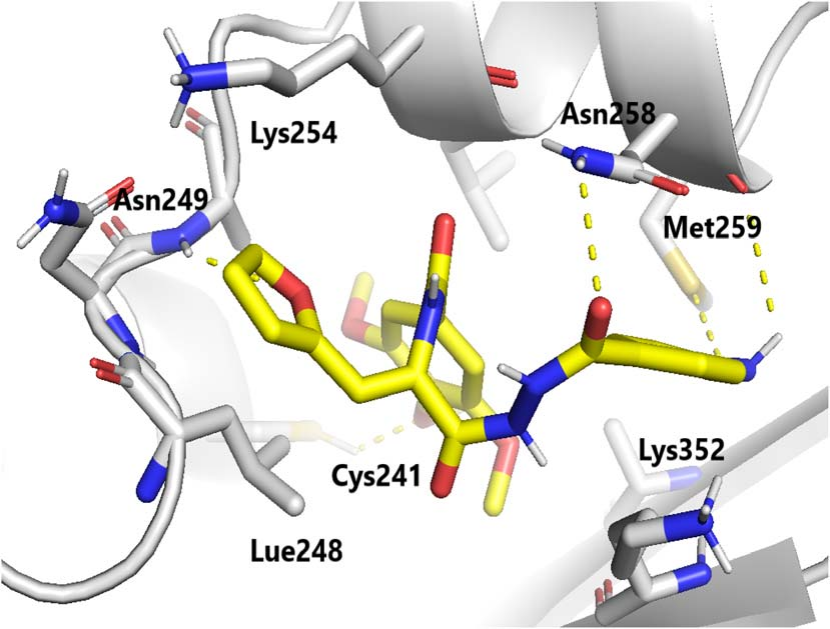
Binding mode of compound 4j in the colchicine-binding pocket of tubulin. Hydrogen bonds are shown using blue dashed lines.

Molecular docking studies were performed to elucidate the superior cytotoxicity and tubulin inhibitory activity of the PABA–acrylamide–CA-4 hybrids, 4a and 4j, compared to CA-4. Notably, CA-4, 4a, and 4j were found to be superimposed and optimally positioned within the colchicine binding site of tubulin (Fig. 6), with binding free energies of −6.2, −8.0, and −8.1 kcal mol^−1^, respectively. Detailed analysis revealed that CA-4, 4a, and 4j (Fig. 7–9) established hydrogen bonding interactions with the key amino acid residue Cys241. Additionally, strong hydrophobic interactions with Gln247, Leu248, Ala250, Lys254, Leu255, Asn258, Lys352, and Ala316 were identified within the tubulin binding pocket.^35,36^

This exceptional tubulin-binding inhibitory activity may be attributed to the newly incorporated PABA fragment, which anchors within a hydrophobic pocket flanked by Lys352, Met259, and Asn258. Additionally, strong hydrogen bonding interactions were observed with Asn258 and Met259, further stabilizing the complex. Moreover, the 4-F-phenyl fragment 4a and the 2-furanyl group 4j are positioned within a hydrophobic pocket bordered by Leu248, Asn249, and Lys254. Furthermore, the formation of additional hydrogen bonds with Asn249 and Lys254 contributes to the enhanced stability of these ligand–tubulin complexes. The docking results show that for the PABA–acrylamide–CA-4 hybrids, 4a and 4j, the cytotoxicity against MCF-7 may result from their ability to inhibit tubulin polymerization.

### 2.4. *In Silico* predictive ADME study for synthesized acrylamide–PABA 4a–j

The ADME descriptors tool in Discovery Studio was used to assess the in *s*ilico pharmacokinetic profiles of the synthesized PAPA–acrylamide derivatives 4a–j (Table 2). Important pharmacokinetic factors were anticipated, such as the blood–brain barrier level (BBB level) and aqueous solubility level.^37,38^ The BBB level value of 4 for all synthesized compounds indicated that they were unable to pass through the blood–brain barrier. This finding is beneficial since it may lessen the likelihood of adverse effects involving the central nervous system.

**Table 2:** Anticipated aqueous solubility and BBB penetration of the acrylamide–PABA compounds 4a–j

Comp. no.	Aq. solubility level	BBB level
4a	2	4
4b	2	4
4c	2	4
4d	2	4
4e	2	4
4f	3	4
4g	2	4
4h	1	4
4i	2	4
4j	3	4

Furthermore, the compounds' expected degrees of aqueous solubility ranged from low to good. The most potent antiproliferative candidate in the series, compound 4j, notably showed a good aqueous solubility profile (aq. sol. level: 3). This result is quite noteworthy and consistent with our study's main goal. The parent compound CA-4 is characterized by poor water solubility, which limits its bioavailability and therapeutic performance. In this study, we strategically designed acrylamide–PAPA derivatives by integrating PABA to enhance the polarity and overall solubility. The enhanced water solubility of compound 4j serves as a strong indication that this design approach is effective, potentially overcoming one of the major pharmacokinetic limitations of CA-4.

## 3. Conclusions

The intricate molecular pathways governing β-tubulin-microtubule dynamics and apoptosis are two obvious targets for pharmacological interventions, given their significant significance in the formation and evolution of cancerous tumors. So, focusing on the inhibition of β-tubulin polymerization and apoptosis promotion is a desirable approach for successful cancer therapy. As an approach, a new series of acrylamide–PABA analogs 4a–j have been constructed and tested for their ability to inhibit the MCF-7 breast cancer cell line. The acrylamide–PABA analog 4j was found to have strong cytotoxic action *via* β-tubulin polymerization inhibition and activating apoptosis. The apoptotic effect of the most active analog was extensively investigated and showed a marked increase in p53 and Bax levels up to 5.14- and 8.36-fold, respectively, accompanied by down-regulation of Bcl-2 to 0.29-fold relative to the controls. Furthermore, the influence of compound 4j on *c*aspase 9 was evaluated. It was found to enhance its level by 9.89-fold in comparison to the untreated controls. The effect of compound 4j on the cell cycle was also examined. Results revealed that compound 4j causes the MCF-7 cells to arrest at the G2/M phase compared to the untreated controls.

## 4. Experimental

### 4.1. Chemistry

#### 4.1.1. General method for the synthesis of (*Z*)-*N*-(3-(2-(4-aminobenzoyl)hydrazineyl)-1-aryl-3-oxoprop-1-en-2-yl)-3,4,5-trimethoxybenzamides 4a–j

A mixture of 4-aminobenzohydrazide (0.0028 mol, 0.42 g) and appropriate ethyl 3-arylacrylate (0.0028 mol) in pure ethanol (20 mL) and glacial acetic acid (2 mL) was refluxed for 10–12 h. The reaction mixture was cooled and poured into ice-cold water once the reaction was finished. The acrylamide–PABA analogs 4a–j were achieved by filtering and crystallizing the separated solid from the alcohol.

##### 4.1.1.1. (*Z*)-*N*-(3-(2-(4-Aminobenzoyl)hydrazineyl)-1-(4-fluorophenyl)-3-oxoprop-1-en-2-yl)-3,4,5-trimethoxybenzamide (4a)

Yield: 72%, m.p. 228–230 °C. ^1^H-NMR (400 MHz, DMSO-*d*_6_) *δ*: 10.03 (s, 1H, NH), 9.96 (s, 1H, NH), 9.94 (s, 1H, NH), 7.68 (d, *J* = 9.1 Hz, 2H), 7.65 (d, *J* = 3.2 Hz, 2H), 7.36 (s, 2H), 7.31 (s, 1H, olefinic CH), 7.25 (t, *J* = 8.9 Hz, 2H), 6.56 (d, *J* = 8.7 Hz, 2H), 5.70 (s, 2H, NH_2_), 3.85 (s, 6H, 2OCH_3_), 3.74 (s, 3H, OCH_3_). ^13^C-NMR (101 MHz, DMSO) *δ*: 166.06, 165.87, 165.28, 161.24, 153.00, 152.56, 140.85, 132.14, 132.06, 131.12, 129.61, 129.44, 129.19, 129.01, 128.86, 119.59, 116.17, 113.01, 106.10, 60.57, 56.53. Analysis: Calc. for C_26_H_25_FN_4_O_6_ (508.51): C 61.41, H 4.96, N 11.02%, found: C 61.52, H 5.07, N 10.89%.

##### 4.1.1.2. (*Z*)-*N*-(3-(2-(4-Aminobenzoyl)hydrazineyl)-1-(4-chlorophenyl)-3-oxoprop-1-en-2-yl)-3,4,5-trimethoxybenzamide (4b)

Yield: 74%, m.p. 239–241 °C. ^1^H-NMR (400 MHz, DMSO-*d*_6_) *δ*: 10.07 (s, 1H, NH), 9.96 (s, 2H, NH_2_), 7.68–7.64 (m, 2H), 7.64–7.60 (m, 2H), 7.57–7.52 (m, 2H), 7.36 (s, 2H), 7.27 (s, 1H, olefinic CH), 6.59–6.55 (m, 2H), 5.71 (s, 2H, NH_2_), 3.86 (s, 6H, 2OCH_3_), 3.74 (s, 3H s, 3H, OCH_3_). ^13^C-NMR (101 MHz, DMSO) *δ*: 166.90, 166.07, 165.21, 153.01, 151.98, 140.89, 133.52, 131.55, 130.36, 129.62, 129.12, 128.87, 128.58, 120.41, 119.56, 113.02, 106.11, 60.57, 56.54. Analysis: Calc. for C_26_H_25_ClN_4_O_6_ (524.96): C 59.49, H 4.80, N 10.67%, found: C 59.38, H 4.85, N 10.78%.

##### 4.1.1.3. (*Z*)-*N*-(3-(2-(4-Aminobenzoyl)hydrazineyl)-1-(4-nitrophenyl)-3-oxoprop-1-en-2-yl)-3,4,5-trimethoxybenzamide (4c)

Yield: 76%, m.p. 243–245 °C. ^1^H-NMR (400 MHz, DMSO-*d*_6_) *δ*: 10.12 (s, 2H, 2NH), 10.02 (s, 1H, NH), 8.25 (d, *J* = 8.8 Hz, 2H), 7.84 (d, *J* = 8.6 Hz, 2H), 7.66 (d, *J* = 8.3 Hz, 2H), 7.35 (s, 2H), 7.31 (s, 1H, olefinic CH), 6.57 (d, *J* = 8.6 Hz, 2H), 5.72 (s, 2H, NH_2_), 3.85 (s, 6H, 2OCH_3_), 3.74 (s, 3H, OCH_3_). ^13^C-NMR (101 MHz, DMSO) *δ*: 166.89, 165.84, 164.99, 153.03, 151.98, 147.15, 141.62, 133.07, 130.74, 129.64, 128.87, 126.75, 124.18, 120.40, 119.47, 113.02, 106.18, 60.58, 56.56. Analysis: Calc. for C_26_H_25_N_5_O_8_ (535.51): C 58.32, H 4.71, N 13.08%, found: C 58.43, H 4.58, N 12.98%.

##### 4.1.1.4. (*Z*)-*N*-(3-(2-(4-Aminobenzoyl)hydrazineyl)-3-oxo-1-(*p*-tolyl)prop-1-en-2-yl)-3,4,5-trimethoxybenzamide (4d)

Yield: 68%, m.p. 237–239 °C. ^1^H-NMR (400 MHz, DMSO-*d*_6_) *δ*: 9.97 (s, 2H, 2NH), 9.90 (s, 1H, NH), 7.66 (d, *J* = 8.2 Hz, 2H), 7.51 (d, *J* = 7.8 Hz, 2H), 7.38 (s, 2H), 7.29 (s, 1H, olefinic CH), 7.21 (d, *J* = 7.8 Hz, 2H), 6.57 (d, *J* = 8.3 Hz, 2H), 5.70 (s, 2H, NH_2_), 3.86 (s, 6H, 2OCH_3_), 3.74 (s, 3H, OCH_3_), 2.30 (s, 3H, CH_3_). ^13^C-NMR (101 MHz, DMSO) *δ*: 166.09, 165.85, 165.42, 152.99, 152.55, 140.79, 139.08, 131.69, 130.38, 129.97, 129.66, 129.61, 129.33, 128.76, 119.60, 113.02, 106.08, 60.58, 56.53, 21.37. Analysis: Calc. for C_27_H_28_N_4_O_6_ (504.54): C 64.28, H 5.59, N 11.10%, found: C 64.44, H 5.71, N 11.01%.

##### 4.1.1.5. (*Z*)-*N*-(3-(2-(4-Aminobenzoyl)hydrazineyl)-1-(4-methoxyphenyl)-3-oxoprop-1-en-2-yl)-3,4,5-trimethoxybenzamide (4e)

Yield: 73%, m.p. 226–228 °C. ^1^H-NMR (400 MHz, DMSO-*d*_6_) *δ*: 10.01 (s, 1H, 2NH), 9.96 (s, 1H, 2NH), 9.87 (s, 1H, NH), 7.65 (d, *J* = 8.3 Hz, 2H), 7.58 (d, *J* = 8.4 Hz, 2H), 7.38 (s, 2H), 7.30 (s, 1H, olefinic CH), 6.96 (d, *J* = 8.4 Hz, 2H), 6.56 (d, *J* = 8.3 Hz, 2H), 5.69 (s, 2H, NH_2_), 3.86 (s, 6H, 2OCH_3_), 3.76 (s, 3H, OCH_3_), 3.74 (s, 3H, OCH_3_). ^13^C-NMR (101 MHz, DMSO) *δ*: 166.08, 165.83, 165.50, 160.21, 152.99, 152.54, 140.77, 131.72, 130.43, 129.60, 129.39, 127.33, 126.95, 119.66, 114.57, 113.02, 106.09, 60.57, 56.53, 55.69. Analysis: Calc. for C_27_H_28_N_4_O_7_ (520.54): C 62.30, H 5.42, N 10.76%, found: C 62.47, H 5.28, N 10.61%.

##### 4.1.1.6. (*Z*)-*N*-(3-(2-(4-Aminobenzoyl)hydrazineyl)-1-(4-(dimethylamino)phenyl)-3-oxoprop-1-en-2-yl)-3,4,5-trimethoxybenzamide (4f)

Yield: 77%, m.p. 215–217 °C. ^1^H-NMR (400 MHz, DMSO-*d*_6_) *δ*: 9.89 (s, 1H, NH), 9.79 (s, 1HNH), 9.78 (s, 1H, NH), 7.65 (d, *J* = 8.3 Hz, 2H), 7.48 (d, *J* = 8.5 Hz, 2H), 7.41 (s, 2H), 7.30 (s, 1H, olefinic CH), 6.70 (d, *J* = 8.6 Hz, 2H), 6.56 (d, *J* = 8.2 Hz, 2H), 5.69 (s, 2H, NH_2_), 3.87 (s, 6H, 2OCH_3_), 3.75 (s, 3H, OCH_3_), 2.93 (s, 6H, N(CH_3_)_2_). ^13^C-NMR (101 MHz, DMSO) *δ*: 166.05, 165.72, 165.71, 152.49, 151.06, 140.66, 135.26, 131.68, 129.58, 127.52, 124.48, 121.77, 119.74, 113.00, 112.15, 106.08, 105.06, 60.57, 56.53, 40.15. Analysis: Calc. for C_28_H_31_N_5_O_6_ (533.59): C 63.03, H 5.86, N 13.13%, found: C 62.87, H 5.97, N 13.22%.

##### 4.1.1.7. (*Z*)-*N*-(3-(2-(4-Aminobenzoyl)hydrazineyl)-1-(3,4-dimethoxyphenyl)-3-oxoprop-1-en-2-yl)-3,4,5-trimethoxybenzamide (4g)

Yield: 71%, m.p. 211–213 °C. ^1^H-NMR (400 MHz, DMSO-*d*_6_) *δ*: 9.98 (d, *J* = 19.5 Hz, 2H), 9.84 (s, 1H), 7.68 (t, *J* = 4.7 Hz, 2H), 7.65 (s, 1H), 7.55 (d, *J* = 8.3 Hz, 1H), 7.34 (s, 1H, olefinic CH), 7.07 (d, *J* = 8.4 Hz, 1H), 7.00 (s, 2H), 6.57 (d, *J* = 8.4 Hz, 2H), 5.70 (s, 2H, NH_2_), 3.84 (s, 3H, OCH_3_), 3.82 (s, 3H, OCH_3_), 3.67 (s, 3H, OCH_3_), 3.63 (s, 6H, 2OCH_3_). ^13^C-NMR (101 MHz, DMSO) *δ*: 166.90, 165.92, 165.27, 153.04, 152.54, 151.98, 148.54, 138.51, 130.88, 129.91, 129.61, 128.87, 126.40, 121.94, 119.63, 113.05, 111.73, 111.22, 107.61, 60.52, 56.10, 56.08. Analysis: Calc. for C_28_H_30_N_4_O_8_ (550.57): C 61.08, H 5.49, N 10.18%, found: C 60.98, H 5.34, N 10.29%.

##### 4.1.1.8. (*Z*)-*N*-(3-(2-(4-Aminobenzoyl)hydrazineyl)-1-(3,5-dibromo-4-hydroxyphenyl)3-oxoprop-1-en-2-yl)-3,4,5-trimethoxybenzamide (4h)

Yield: 67%, 238–240 °C. ^1^H-NMR (400 MHz, DMSO-*d*_6_) *δ*: 10.14 (s, 1H, NH), 10.00 (d, *J* = 12.1 Hz, 1H, NH), 9.91 (d, *J* = 22.6 Hz, 1H, NH), 7.99 (s, 1H, OH), 7.84 (s, 2H, Ar–H), 7.64 (dd, *J* = 8.6, 2.6 Hz, 2H, Ar–H), 7.32 (s, 2H, Ar–H), 7.29 (d, *J* = 14.2 Hz, 1H, olefinic CH), 6.61–6.50 (m, 2H, Ar–H), 5.70 (s, 2H, NH_2_), 3.86 (s, 6H, 2OCH_3_), 3.73 (s, 3H, OCH_3_). ^13^C-NMR (101 MHz, DMSO) *δ*: 165.52, 164.41, 164.12, 152.56, 152.09, 145.18, 140.29, 135.22, 133.25, 132.92, 129.14, 128.73, 119.09, 117.14, 112.53, 111.75, 105.40, 60.15, 56.06. Analysis: Calc. for C_26_H_24_Br_2_N_4_O_7_ (664.31): C 47.01, H 3.64, N 8.43%, found: C 46.86, H 3.76, N 8.59%.

##### 4.1.1.9. (*Z*)-*N*-(3-(2-(4-Aminobenzoyl)hydrazineyl)-3-oxo-1-(3,4,5-trimethoxyphenyl)prop-1-en-2-yl)-3,4,5-trimethoxybenzamide (4i)

Yield: 73%, m.p. 230–232 °C. ^1^H NMR (400 MHz, DMSO-*d*_6_) *δ*: 10.05 (s, 1H, NH), 9.98 (s, 1H, NH), 9.95 (s, 1H, NH), 7.80–7.66 (m, 2H), 7.45 (s, 2H), 7.39 (s, 1H, olefinic CH), 7.02 (s, 2H), 6.59 (d, *J* = 8.1 Hz, 2H), 5.72 (s, 2H), 3.86 (s, 9H, 3OCH_3_), 3.75 (s, 3H, OCH_3_), 3.67 (s, 6H, 2OCH_3_). ^13^C-NMR (101 MHz, DMSO) *δ*: 166.05, 165.83, 165.18, 153.07, 152.96, 152.57, 140.86, 138.61, 132.40, 131.13, 129.63, 129.29, 128.67, 119.63, 113.03, 107.67, 106.06, 60.64, 60.53, 56.55, 56.08. Analysis: Calc. for C_29_H_32_N_4_O_9_ (580.59): C 59.99, H 5.56, N 9.65%, found: C 60.11, H 5.72, N 9.52%.

##### 4.1.1.10. (*Z*)-*N*-(3-(2-(4-Aminobenzoyl)hydrazineyl)-1-(furan-2-yl)-3-oxoprop-1-en-2-yl)-3,4,5-trimethoxybenzamide (4j)

Yield: 75%, m.p. 219–221 °C. ^1^H NMR (400 MHz, DMSO-*d*_6_) *δ*: ^1^H-NMR (400 MHz, DMSO-*d*_6_) *δ*: 10.01 (s, 1H, NH), 9.96 (s, 1H, NH), 9.83 (s, 1H, NH), 7.85–7.76 (m, 1H), 7.65 (d, *J* = 8.2 Hz, 2H), 7.40 (s, 2H), 7.24 (s, 1H, olefinic CH), 6.76 (d, *J* = 3.5 Hz, 1H), 6.64–6.60 (m, 1H), 6.57 (d, *J* = 8.2 Hz, 2H), 5.71 (s, 2H, NH_2_), 3.87 (s, 6H, 2OCH_3_), 3.75 (s, 3H, OCH_3_). ^13^C-NMR (101 MHz, DMSO) *δ*: 166.06, 165.71, 164.68, 152.99, 152.56, 150.06, 145.17, 140.77, 129.61, 129.51, 126.80, 119.57, 118.65, 114.72, 113.02, 112.83, 106.09, 60.58, 56.53. Analysis: Calc. for C_24_H_24_N_4_O_7_ (480.48): C 60.00, H 5.03, N 11.66%, found: C 60.06, H 4.98, N 11.59%.

### 4.2. Biological study

The biological activity studies of the constructed acrylamide–PABA molecules 4a–j were added in the ESI see Appendix A.†

## Data availability

The authors confirm that the data supporting the findings of this study are available within the article and/or its ESI.†

## Conflicts of interest

No potential conflict of interest was reported by the author(s).

## Supplementary Material

RA-015-D5RA02384J-s001
