# Supplementary material for: Design, synthesis and cytotoxic research of a novel antitumor model based on acrylamide–PABA analogs via β-tubulin inhibition
Source: RSC Adv. 2025 Jun 3;15(23):18490–500. doi: 10.1039/d5ra02384j (PMC12131314; doi:10.1039/d5ra02384j)
Supplement: RA-015-D5RA02384J-s001 [file RA-015-D5RA02384J-s001.pdf]

Supplementary Information (SI) for RSC Advances.
This journal is © The Royal Society of Chemistry 2025

Supporting Information

Design, Synthesis and Cytotoxic Research of Novel Antitumor Model Based on Acrylamide-PABA Analogs *via* β-Tubulin Inhibition

Maha Ali Alghamdi [1], Mustafa R. Abdulbaqi [2], Dalal Sulaiman Alshaya [3], Jawaher Alharthi [1],Hanadi A. Katouah [4], Fahmy Gad Elsaid [5], Eman Fayad [1], Ali H. Abu Almaaty [6,*], Abdullah Yahya Abdullah Alzahrani [7] and Botros Y. Beshay [8]

[1] Department of Biotechnology, College of Sciences, Taif University, P.O. Box 11099, Taif 21944, Saudi Arabia; [2] Department of Pharmaceutics, College of Pharmacy, Al-Naji University, Baghdad 10015, Iraq; [3] Department of Biology, College of Science, Princess Nourah bint Abdulrahman University, P.O. Box 84428, Riyadh 11671, Saudi Arabia; [4] Chemistry Department, College of Science, Umm Al-Qura University, 21955, Makkah, Saudi Arabia; [5] Department of Biology, College of Science, King Khalid University, PO Box 960, Asir, Abha, 61421, Saudi Arabia; [6] Department of Zoology, Faculty of Science, Port Said University, Port Said, Egypt; [7]. Faculty of Science and Arts, Mohail Asser, King Khalid University, Saudi Arabia; Pharmaceutical Sciences (Pharmaceutical Chemistry) Department, College of Pharmacy, Arab Academy for Science, Technology and Maritime Transport, Alexandria P.O. Box 1029, Egypt.

** To whome correspondence should be addressed*

Ali H. Abu Almaaty, PhD. Department of Zoology, Faculty of Science, Port Said University, Port Said, Egypt.

E-mail address: aliabuelmaaty8@gmail.com **(Ali H. Abu Almaaty)**

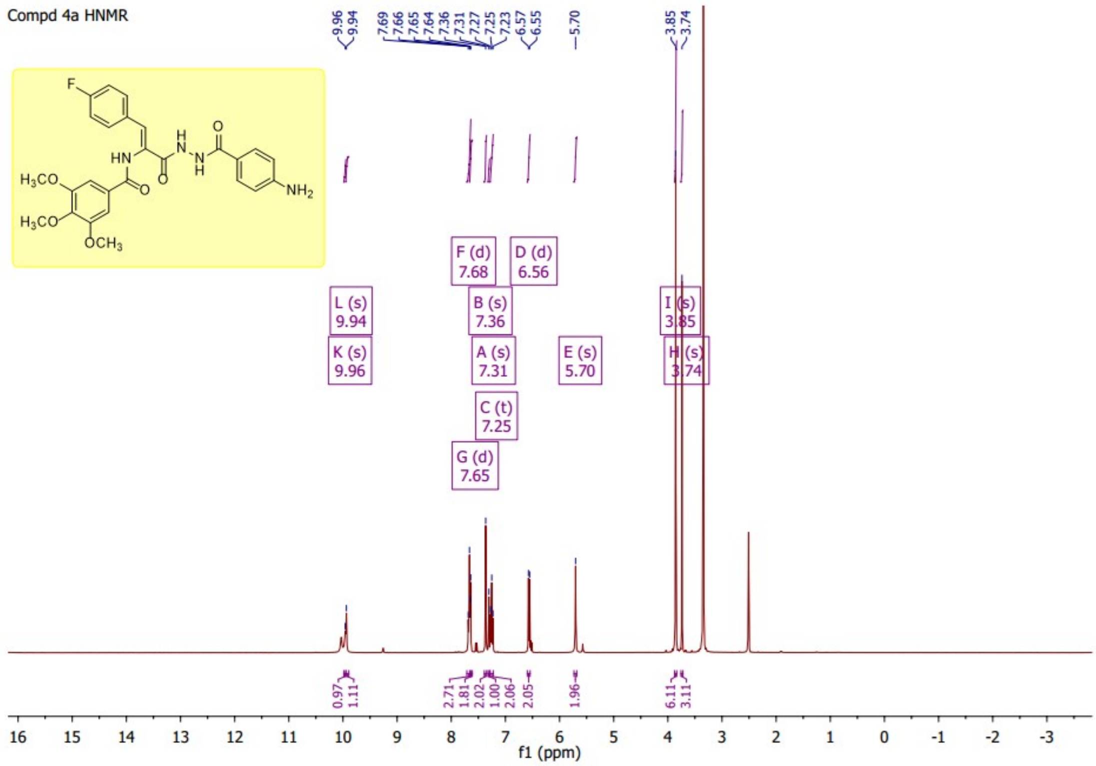

Figure S1: ^{1}H-NMR spectrum of compound **4a**

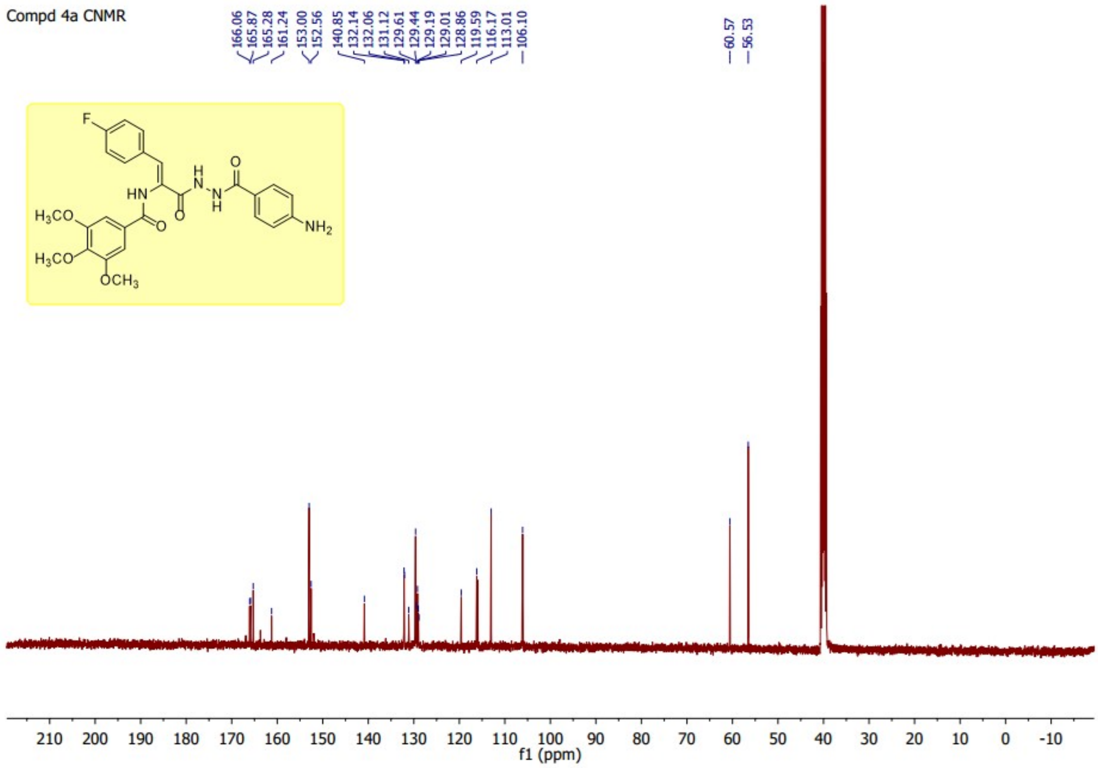

Figure S2: ^{13}C-NMR spectrum of compound **4a**

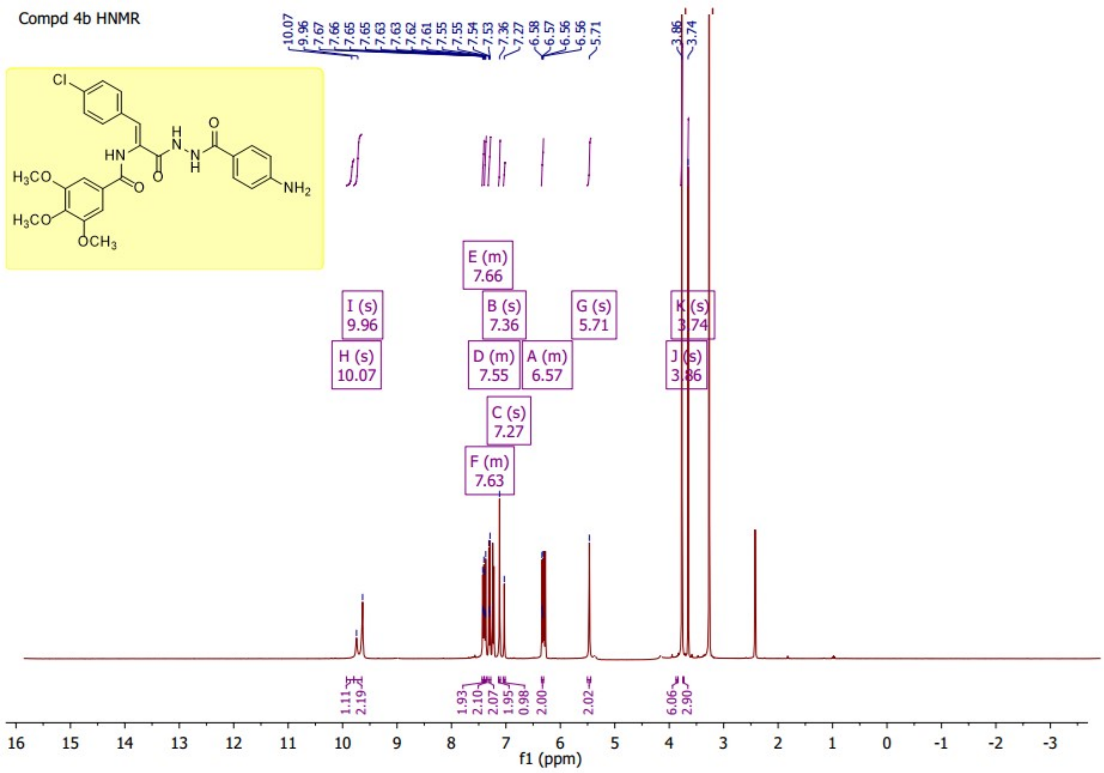

Figure S3: ^{1}H-NMR spectrum of compound **4b**

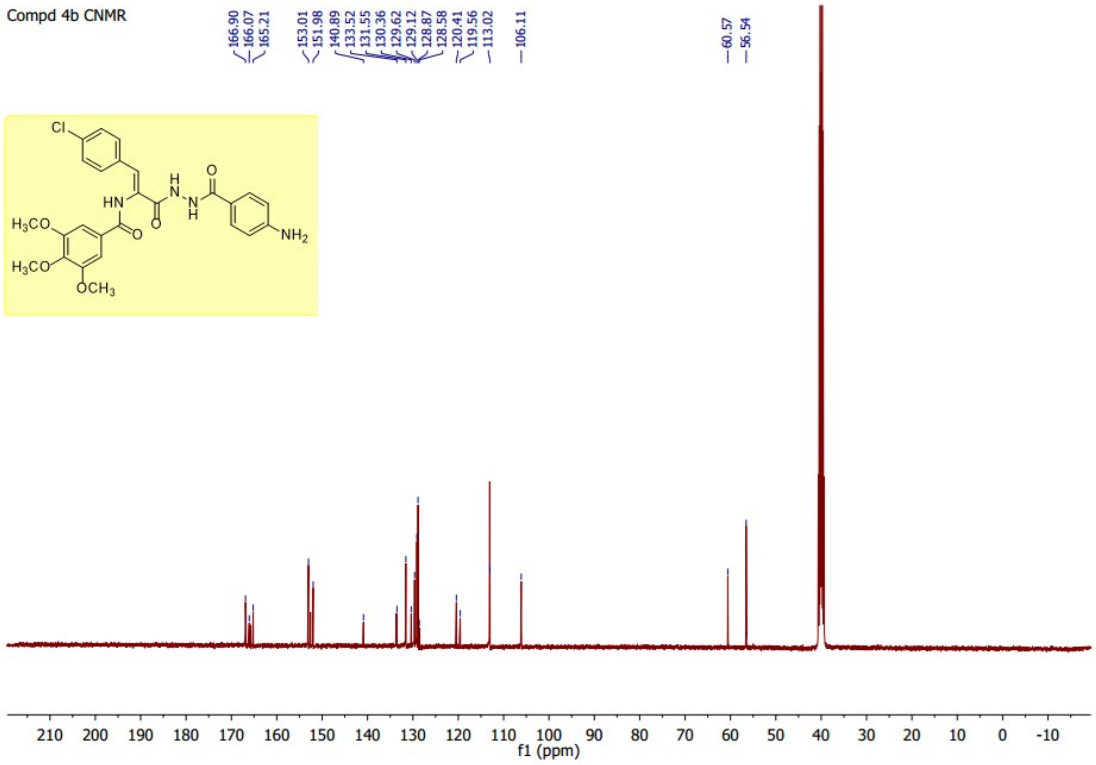

Figure S4: ^{13}C-NMR spectrum of compound **4b**

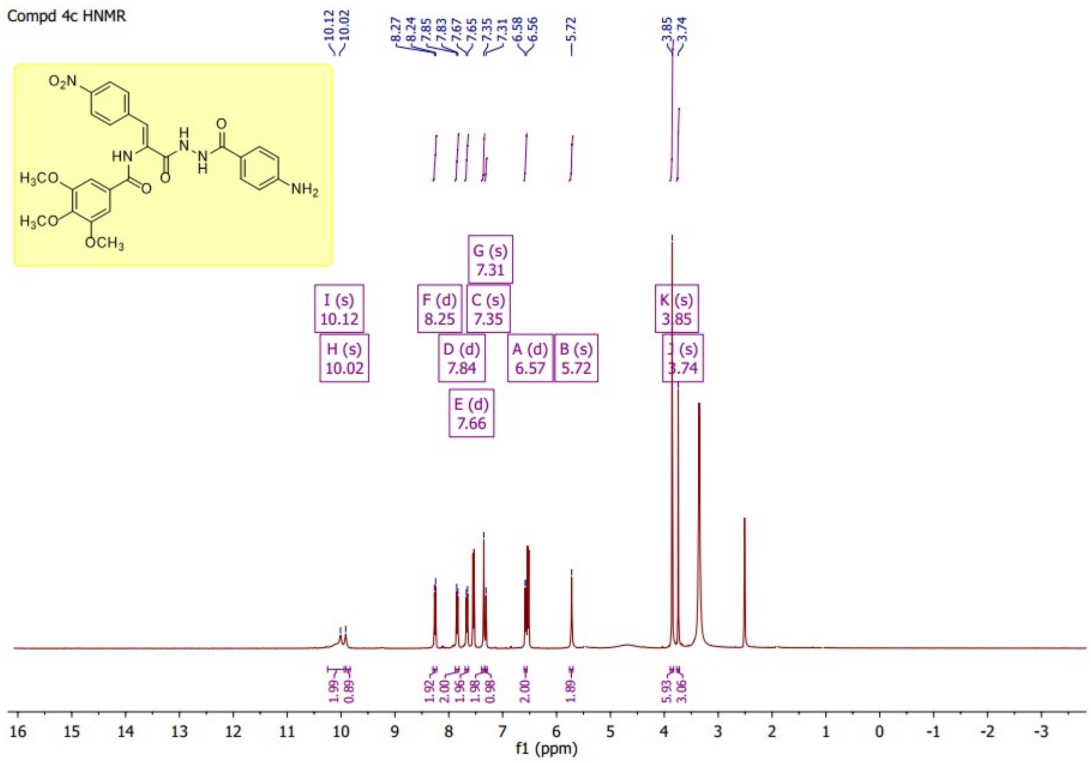

Figure S5: ^{1}H-NMR spectrum of compound **4c**

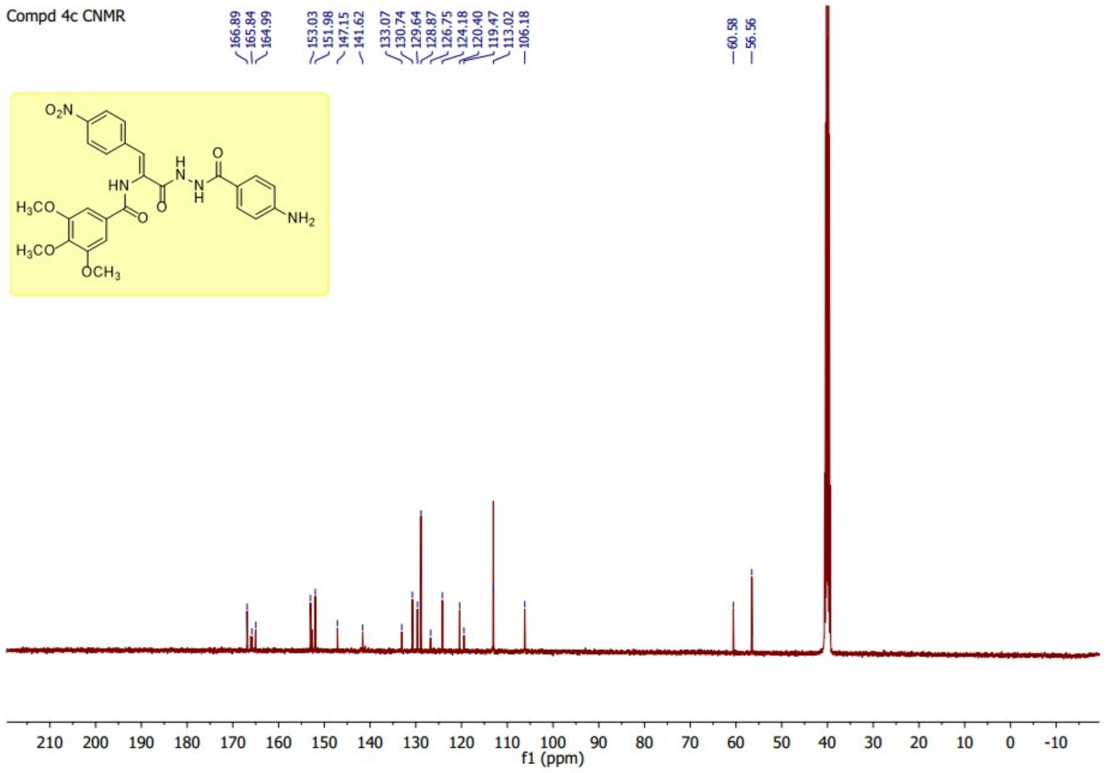

Figure S6: ^{13}C-NMR spectrum of compound **4c**

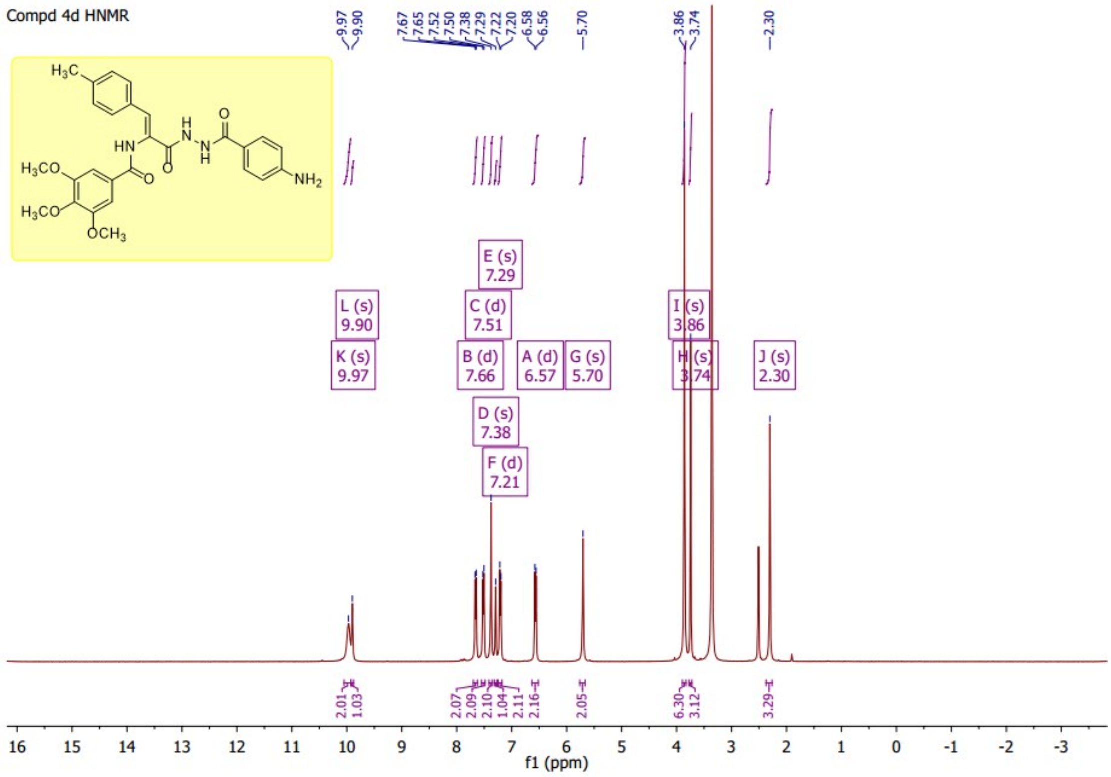

Figure S7: ^{1}H-NMR spectrum of compound **4d**

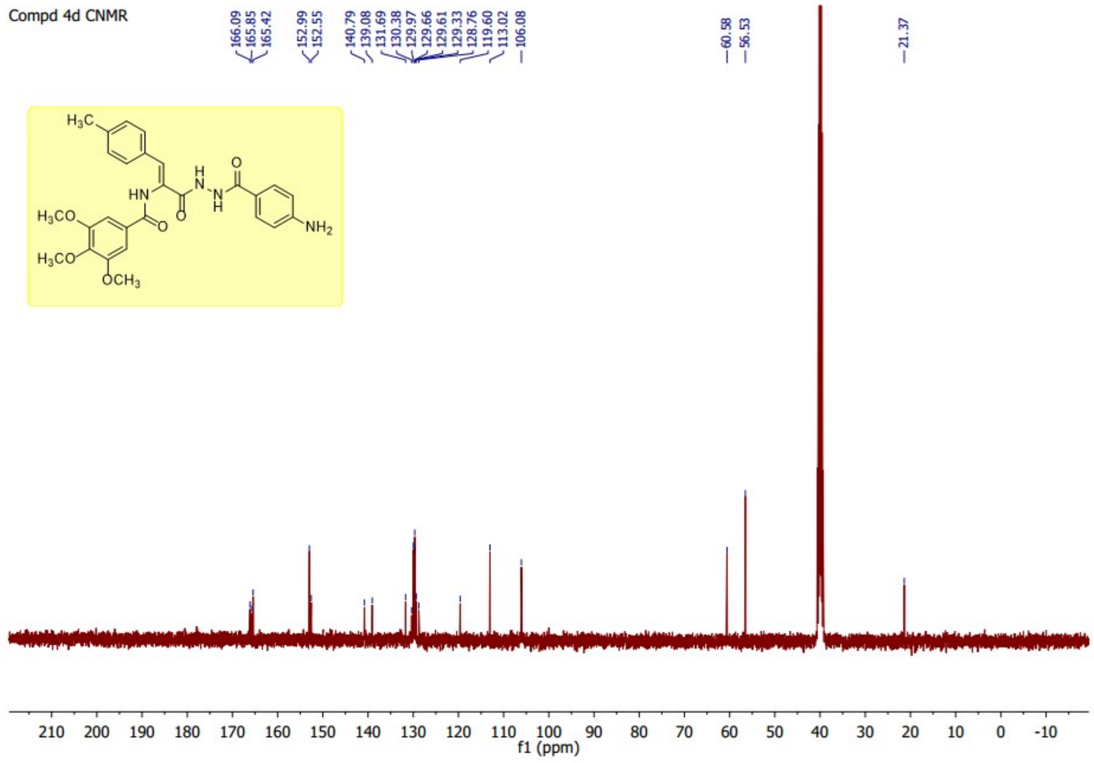

Figure S8: ^{13}C-NMR spectrum of compound **4d**

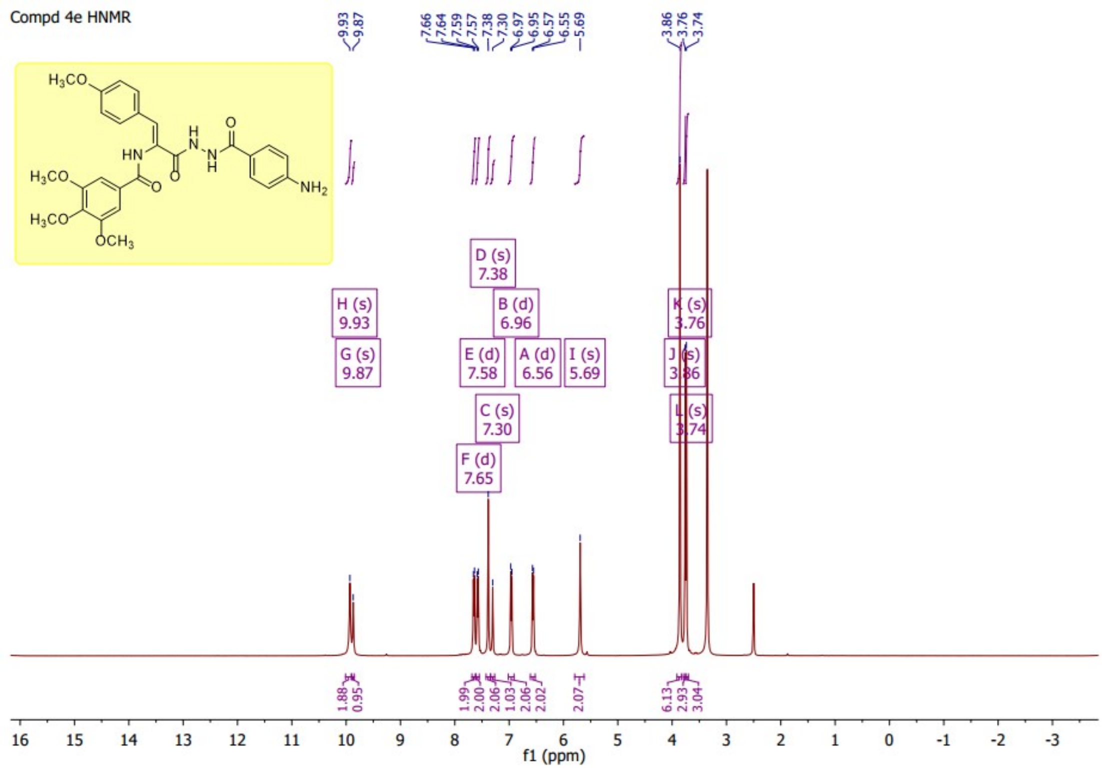

Figure S9: ^{1}H-NMR spectrum of compound **4e**

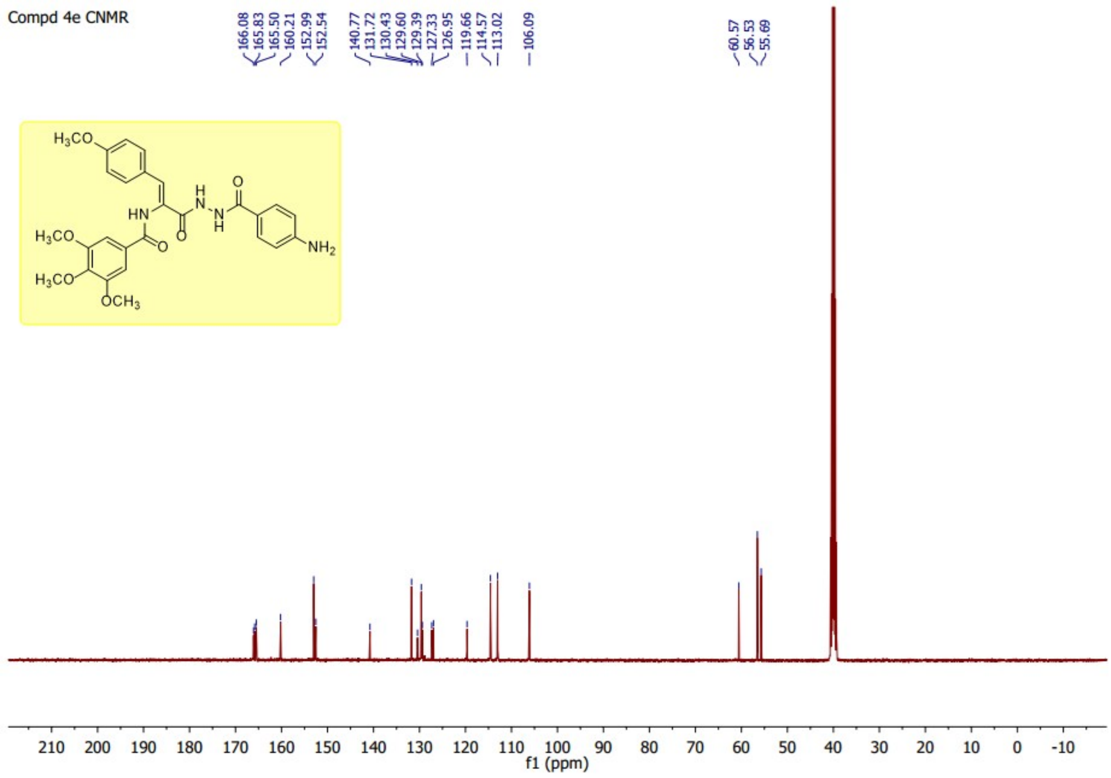

Figure S10: ^{13}C-NMR spectrum of compound **4e**

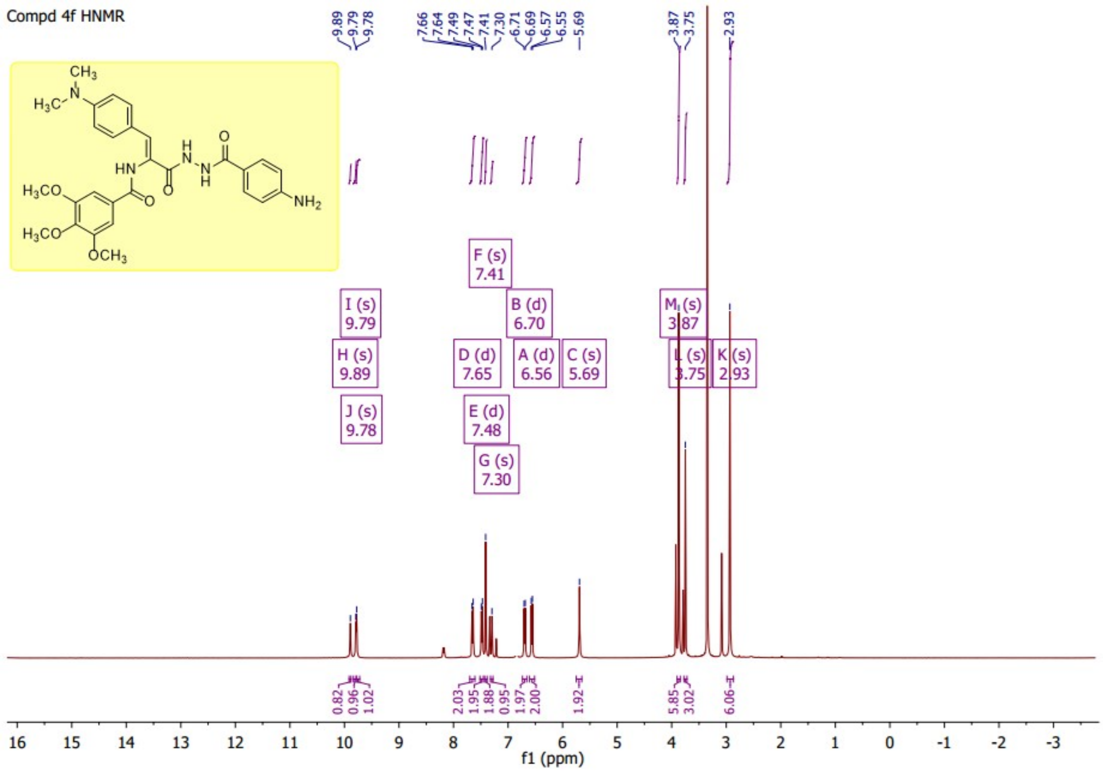

Figure S11: ^{1}H-NMR spectrum of compound **4f**

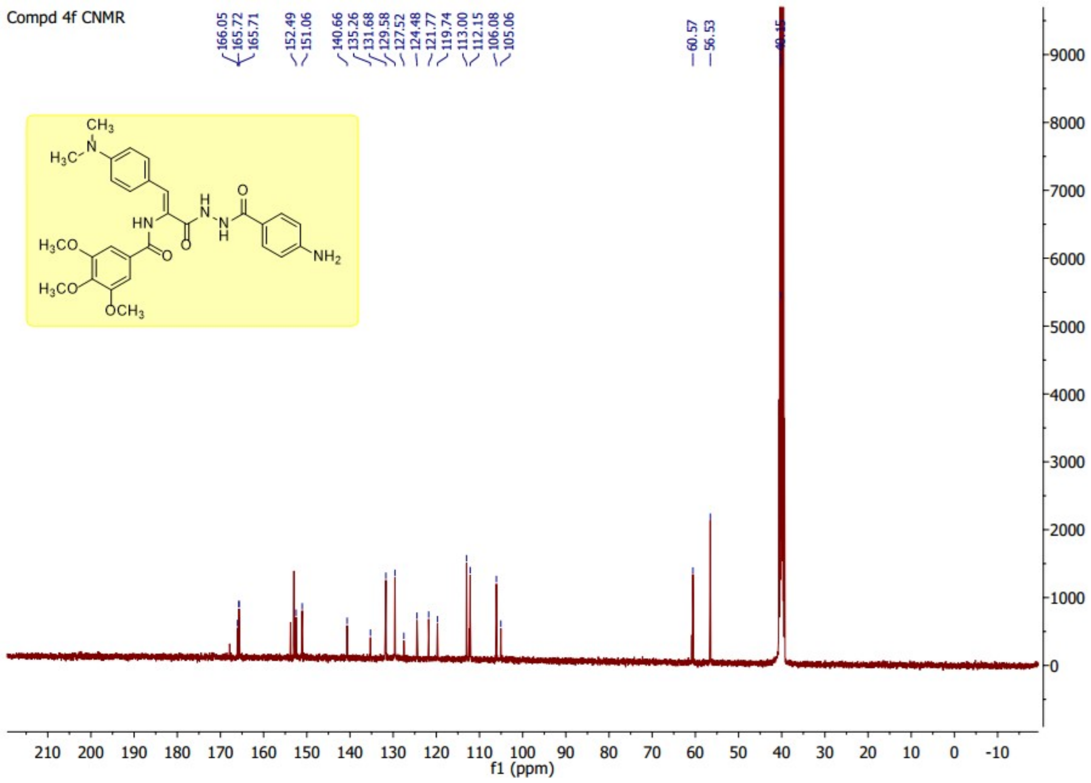

Figure S12: ^{13}C-NMR spectrum of compound **4f**

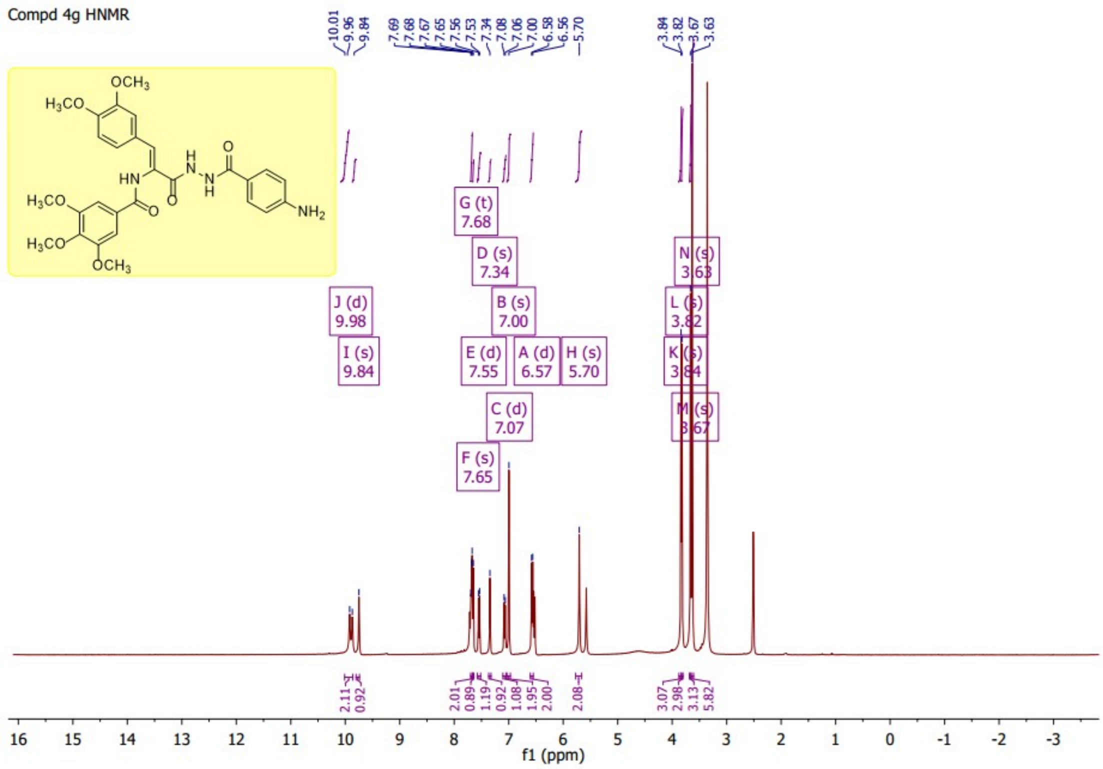

Figure S13: ^{1}H-NMR spectrum of compound **4g**

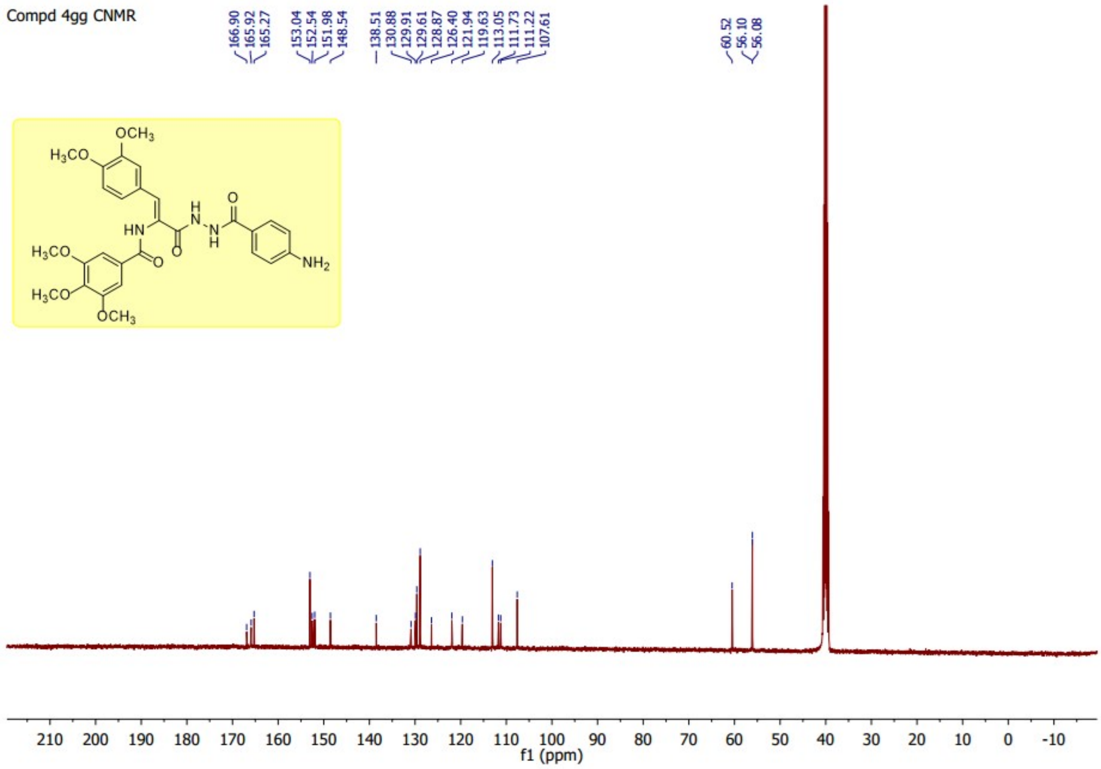

Figure S14: ^{13}C-NMR spectrum of compound **4g**

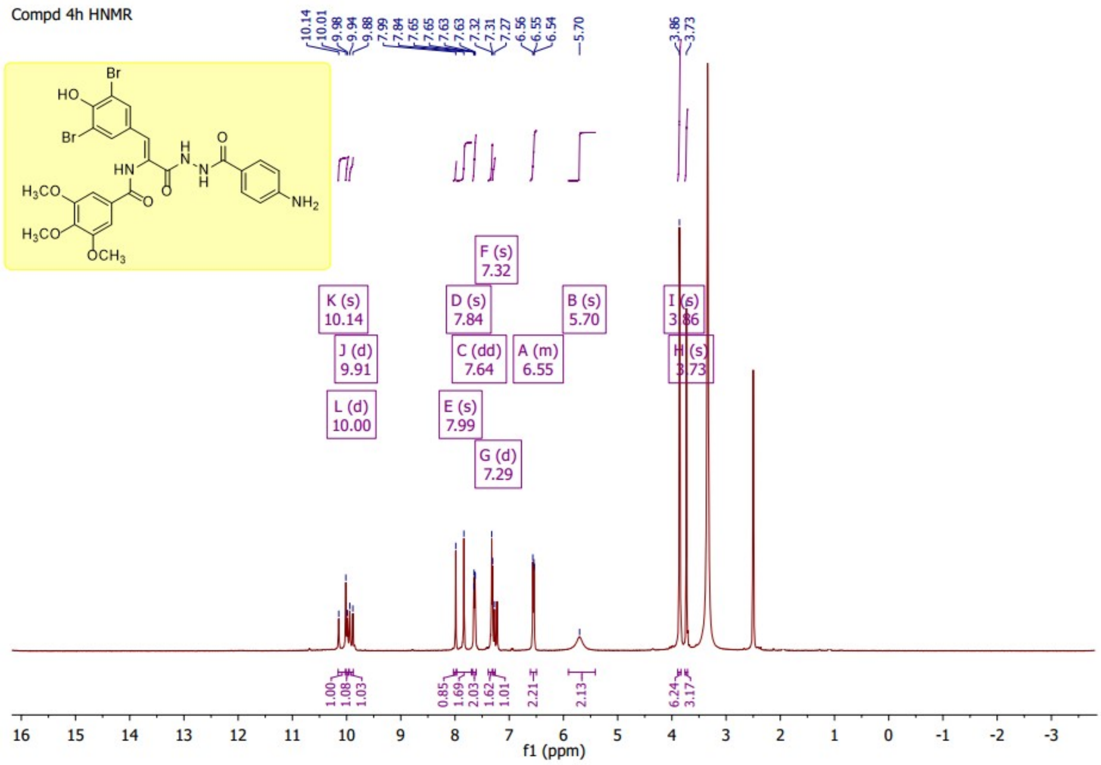

Figure S15: ^{1}H-NMR spectrum of compound **4h**

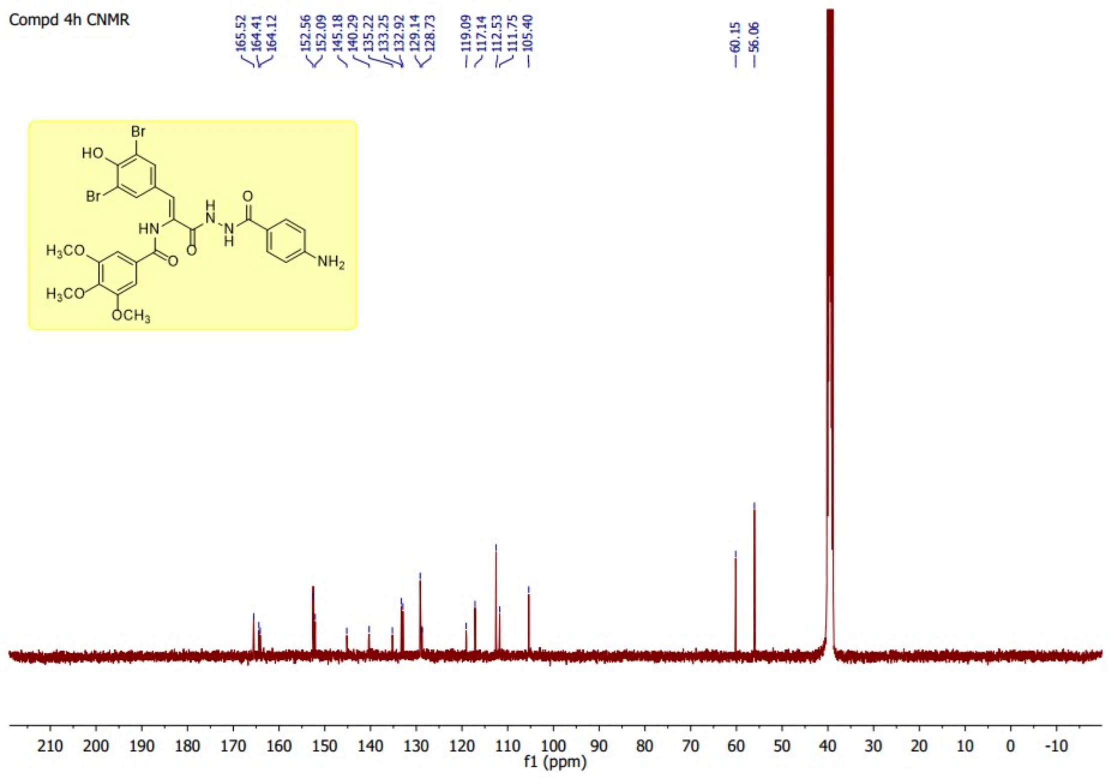

Figure S16: ^{13}C-NMR spectrum of compound **4h**

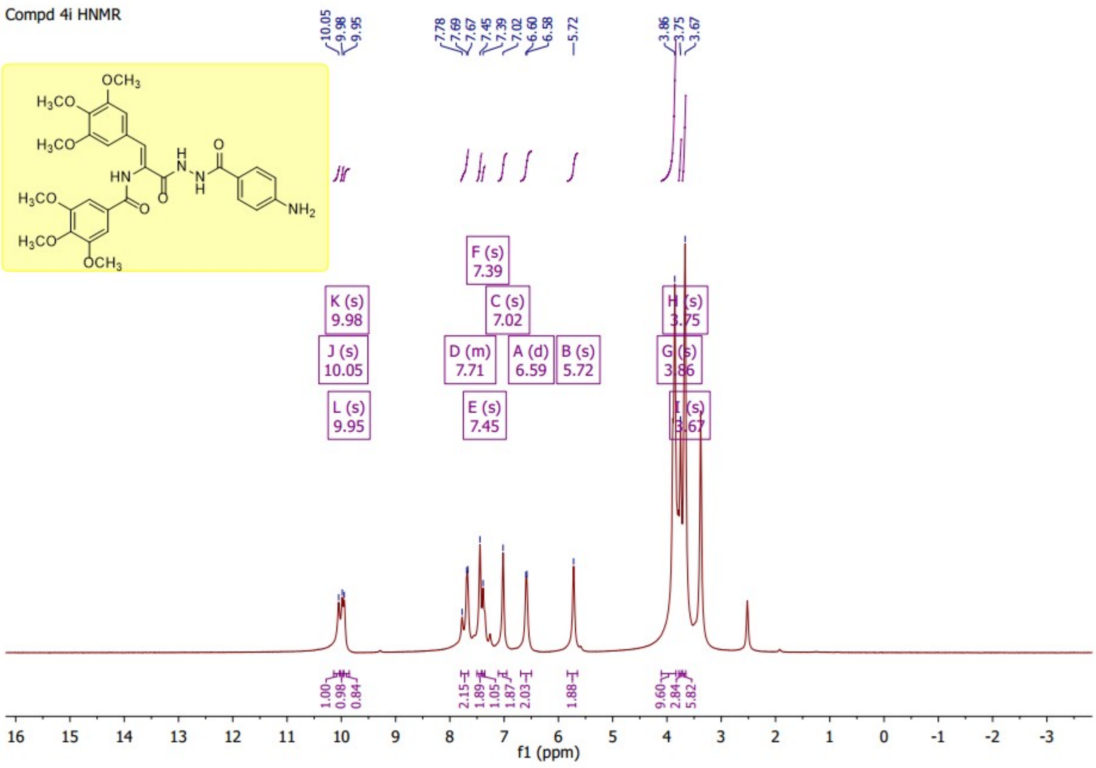

Figure S17: ^{1}H-NMR spectrum of compound **4i**

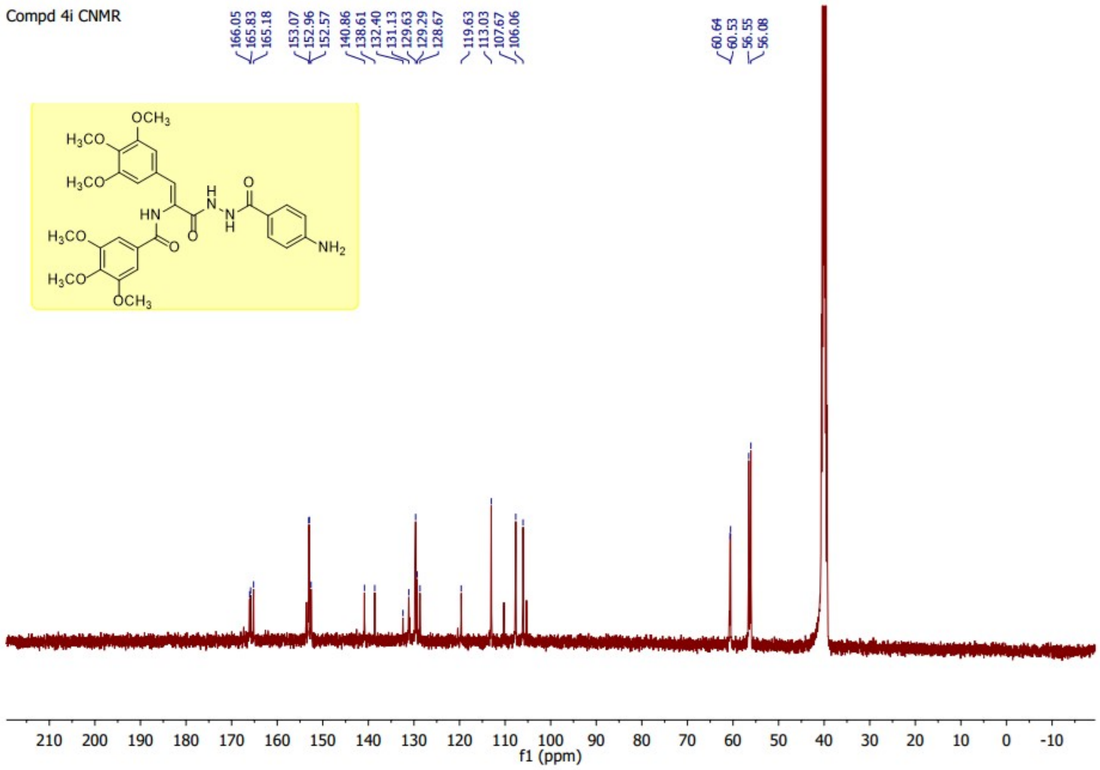

Figure S18: ^{13}C-NMR spectrum of compound **4i**

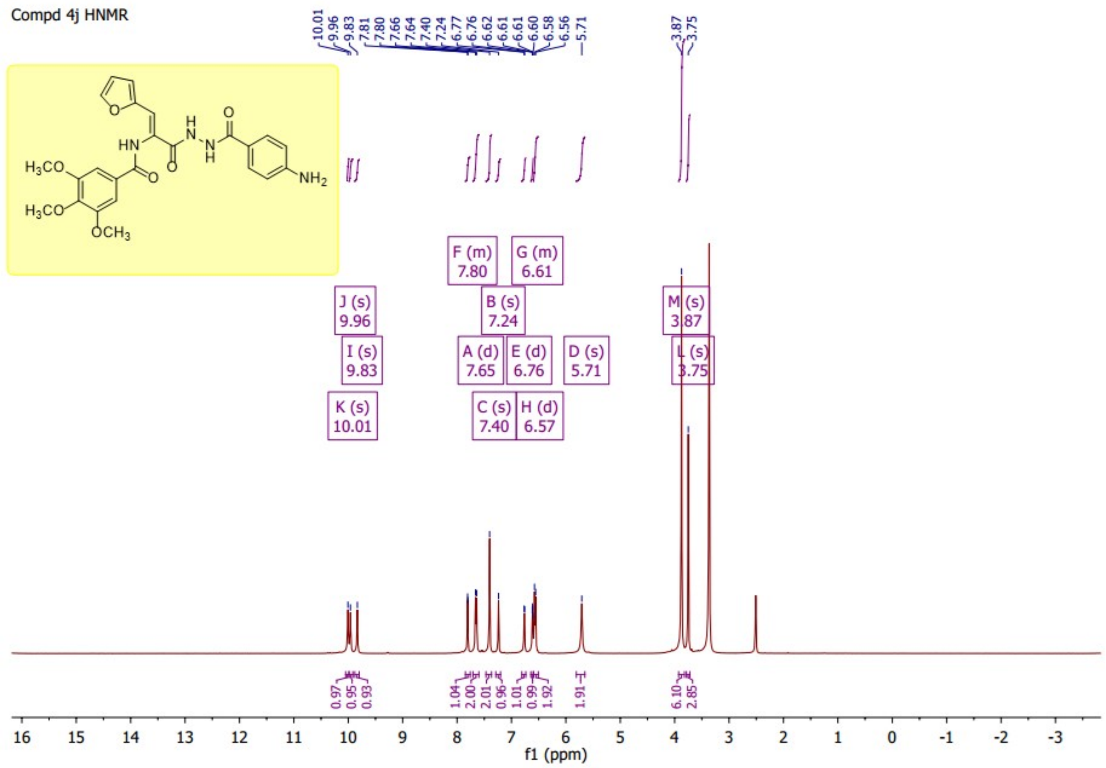

Figure S19: ^{1}H-NMR spectrum of compound **4j**

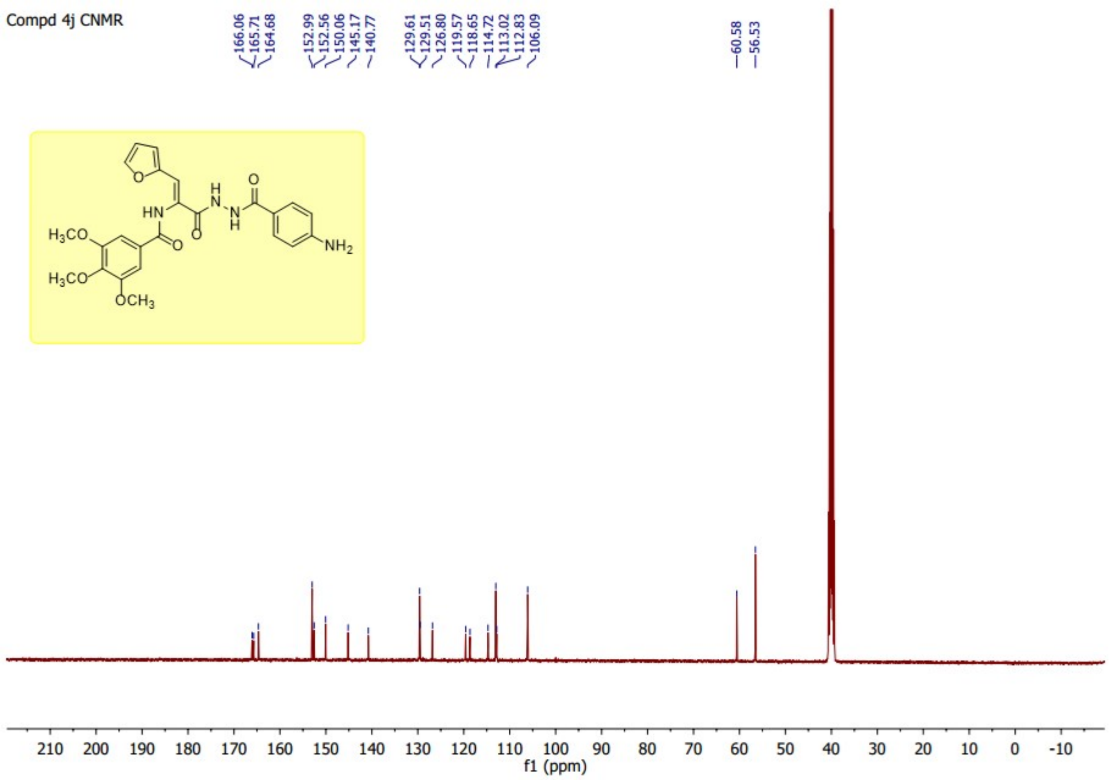

Figure S20: ^{13}C-NMR spectrum of compound **4j**

Appendix A

S4.2. Biological Studies

S4.2.1. Cytotoxic activity evaluation

To measure the cytotoxic actvity of the synthesized acrylamide-PABA derivatives **4a-j** in breast MCF-7 (ATCC Cat. No. HTB-22), liver HepG2 (ATCC Cat No. HB-8065), and normal breast MCF-10A (ATCC Cat. No. CRL 10317) cell lines, cell viability assay was assessed using MTT assay method. Cells at density of 1 x 10^4 were seeded in a 96-well plate at 37 ^{o}C for 24 h under 5% CO_2. After incubation, the cells were treated with different concentrations of the test acrylamide-PABA hybrids **4a-j** and incubated for 24 h, then 20 µl of MTT solution at 5 mg/mL was applied and incubated for 4 h at 37 °C. Dimethyl sulphoxide (DMSO) in volume of 100 µl was added to each well to dissolve the purple formazan that had formed. The color intensity of the formazan product, which represents the growth condition of the cells, is quantified by using an ELISA plate reader (EXL 800, USA) at 570 nm absorbance. The experimental conditions were carried out with at least three replicates, and the experiments were repeated at least three times.

S4.2.2. β-tubulin inhibition Assay

Compounds **4a, 4j** and Col were evaluated for their β-tubulin inhibitory activity according to manufacturer's instructions

ab245722
Human Beta-Tubulin
SimpleStep ELISA® Kit

For the quantitative measurement of Beta-Tubulin in human cell and tissue homogenate extract samples.

This product is for research use only and is not intended for diagnostic use.

S4.2.3. Apoptosis assay for compound 4j

Apoptosis in MCF-7 cells was investigated using fluorescent Annexin V-FITC/ PI detection kit (*BioVision* Annexin V-FITC Apoptosis Detection Kit, Catalog #: K101) by flow cytometry assay. MCF-7 cells at a density of 2×10^5 per well were treated with compound **4j** at the IC_{50} (μM) for 48 h, then the cells were harvested and stained with Annexin V-FITC/ PI dye for 15 min in the dark at 37 °C. The samples were immediately analyzed using *FACS Calibur* flow cytometer (Becton and Dickinson, Heidelberg, Germany).

S4.2.4. qRT-PCR measurements of p53, Bax, Bcl2 and *c*aspase 9 for compound 4j

Real-time PCR for p53, BAX, Bcl-2 and *c*aspase 9 genes expression was done using commercial Qiagen RNA extraction/ BioRad SYBER green PCR master mix according manufacturer's instructions. Briefly, 2 µl of cDNA template, 10 pMol of each forward and reverse primer, 10 µl of 2X Master Mix and to 20 µl total reaction mixture volume by nuclease free water and then was introduced to thermal cycler

instrument (Thermo Scientific, USA). The cycling parameters for the PCR amplification were achieved by initial denaturation at 95 °C for 3 minutes followed by 40 cycles of 94 °C for 15 seconds and annealing/extension step at 60 °C for 1 min. Relative quantification of target genes was run on Rotor-Gene 6000 Series Software 1.7 (Build 87).

Table 1: The primer sequences for Real Time PCR assay

Gene	Sequences (5′-3′)
**P53**	F: AGAGTCTATAGGCCCACCCC<br>R: GCTCGACGCTAGGATCTGAC
**Bax**	F: GAGGAACTGGACAGTAACATGGAGCT<br>R: CGGCCCCAGTTGAAGTTGC
**Bcl2**	F: GCCGGTTCAGGTACTCAGTCATC<br>R: GTCACCTTCACCGTTCCA
**GAPDH**	F: GCACCGTCAAGGCTGAGAAC<br>R: ATGGTGGTGAAGACGCCAGT

S4.2.5. Cell cycle analysis of compound 4j

Cell cycle analysis in MCF-7 cells was investigated using fluorescent Annexin V-FITC/ PI detection kit (*BioVision* EZCell™ Cell Cycle Analysis Kit Catalog #K920) by flow cytometry assay. MCF-7 cells at a density of 2×10^5 per well were harvested and washed twice in PBS. After that, the cells were incubated at 37 °C and 5% CO_2. The medium was incubated with the tested compound **4j** at the IC_{50} (μM) for 48 h, washed twice in PBS, fixed with 70% ethanol, rinsed again with PBS. Afterward, medium was stained with DNA fluorochrome PI for 15 min at 37 °C. The samples were immediately analyzed using Facs Calibur flow cytometer (Becton and Dickinson, Heidelberg, Germany).

S4.3. Molecular modeling study

Molecular docking simulations were conducted using AutoDock Vina to investigate ligand interactions with the tubulin complex. The three-dimensional (3D) structure of the tubulin heterodimer was obtained from the Protein Data Bank (PDB ID: 1SA0), with chain B of the colchicine-bound complex selected as the docking target. Ligand structures were sketched and subjected to energy minimization, while protein

preparation was carried out using the Discovery Studio Suite (v5.2). 3D figures were generated using Pymol software.
